# Supplementary material for: Leishmaniasis in Eurasia and Africa: geographical distribution of vector species and pathogens
Source: R Soc Open Sci. 2019 May 22;6(5):190334. doi: 10.1098/rsos.190334 (PMC6549972; doi:10.1098/rsos.190334)
Supplement: Suppelentary material part I [file rsos190334supp1.pdf]

Supplementary material

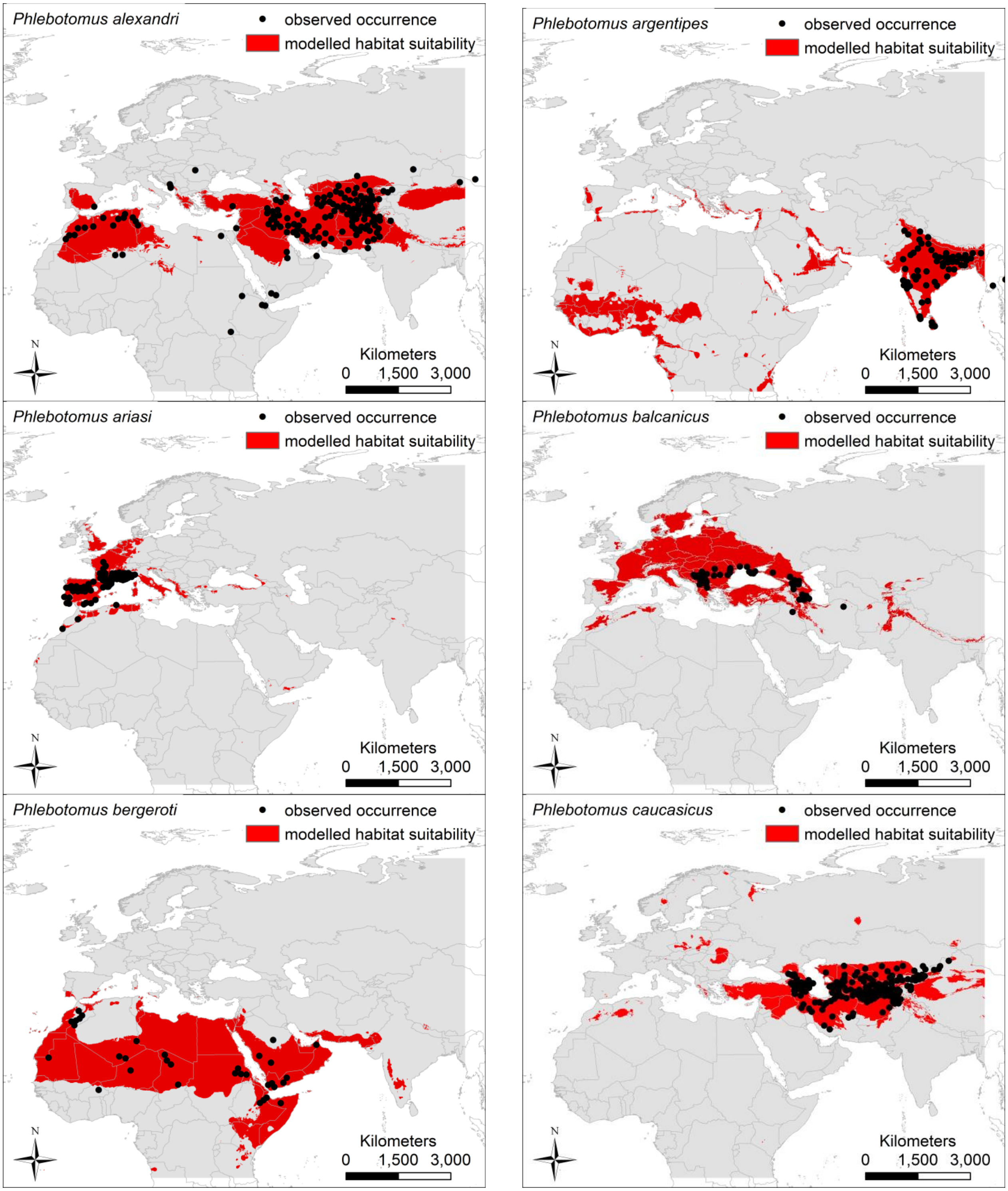

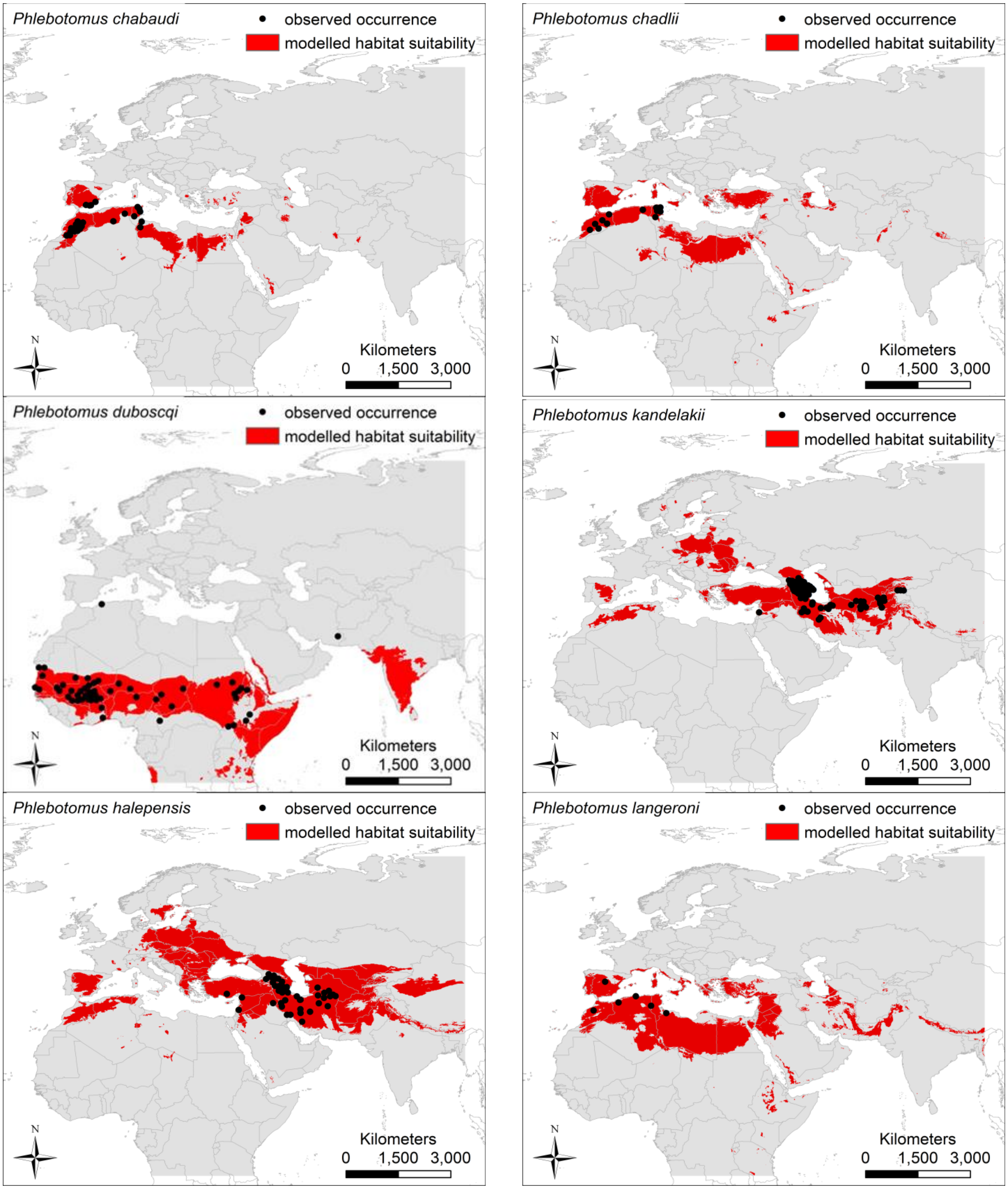
Phlebotomus chabaudi
observed occurrence
modelled habitat suitability
Kilometers
0 1,500 3,000
Phlebotomus chadlii
observed occurrence
modelled habitat suitability
Kilometers
0 1,500 3,000
Phlebotomus duboscqi
observed occurrence
modelled habitat suitability
Kilometers
0 1,500 3,000
Phlebotomus kandelakii
observed occurrence
modelled habitat suitability
Kilometers
0 1,500 3,000
Phlebotomus halepensis
observed occurrence
modelled habitat suitability
Kilometers
0 1,500 3,000
Phlebotomus langeroni
observed occurrence
modelled habitat suitability
Kilometers
0 1,500 3,000

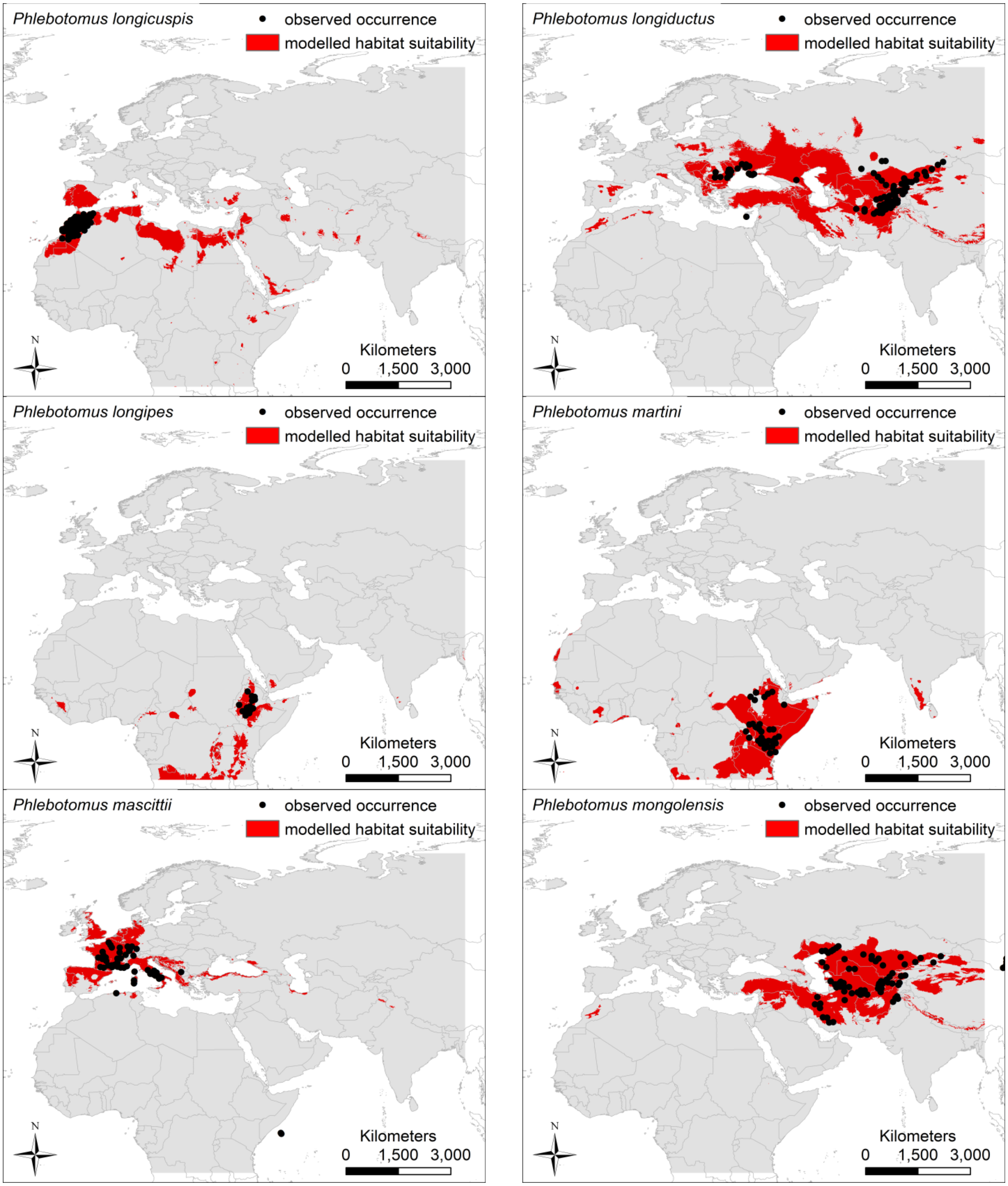
Phlebotomus longicuspis
observed occurrence
modelled habitat suitability
N
Kilometers
0 1,500 3,000
Phlebotomus longiductus
observed occurrence
modelled habitat suitability
N
Kilometers
0 1,500 3,000
Phlebotomus longipes
observed occurrence
modelled habitat suitability
N
Kilometers
0 1,500 3,000
Phlebotomus martini
observed occurrence
modelled habitat suitability
N
Kilometers
0 1,500 3,000
Phlebotomus mascittii
observed occurrence
modelled habitat suitability
N
Kilometers
0 1,500 3,000
Phlebotomus mongolensis
observed occurrence
modelled habitat suitability
N
Kilometers
0 1,500 3,000

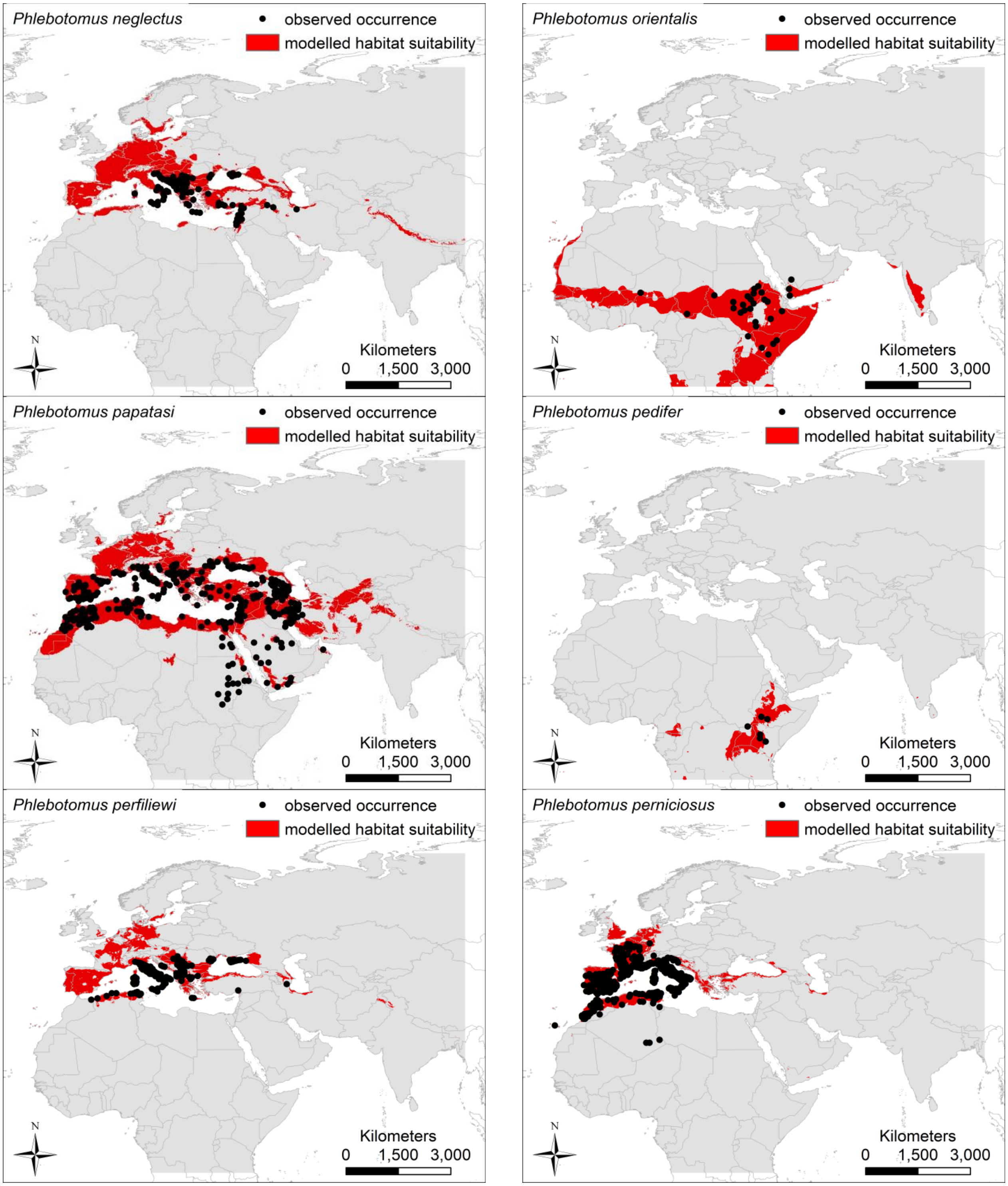

Phlebotomus neglectus
observed occurrence
modelled habitat suitability
Kilometers
0 1,500 3,000
N
Phlebotomus orientalis
observed occurrence
modelled habitat suitability
Kilometers
0 1,500 3,000
N
Phlebotomus papatasi
observed occurrence
modelled habitat suitability
Kilometers
0 1,500 3,000
N
Phlebotomus pedifer
observed occurrence
modelled habitat suitability
Kilometers
0 1,500 3,000
N
Phlebotomus perfiliewi
observed occurrence
modelled habitat suitability
Kilometers
0 1,500 3,000
N
Phlebotomus perniciosus
observed occurrence
modelled habitat suitability
Kilometers
0 1,500 3,000
N

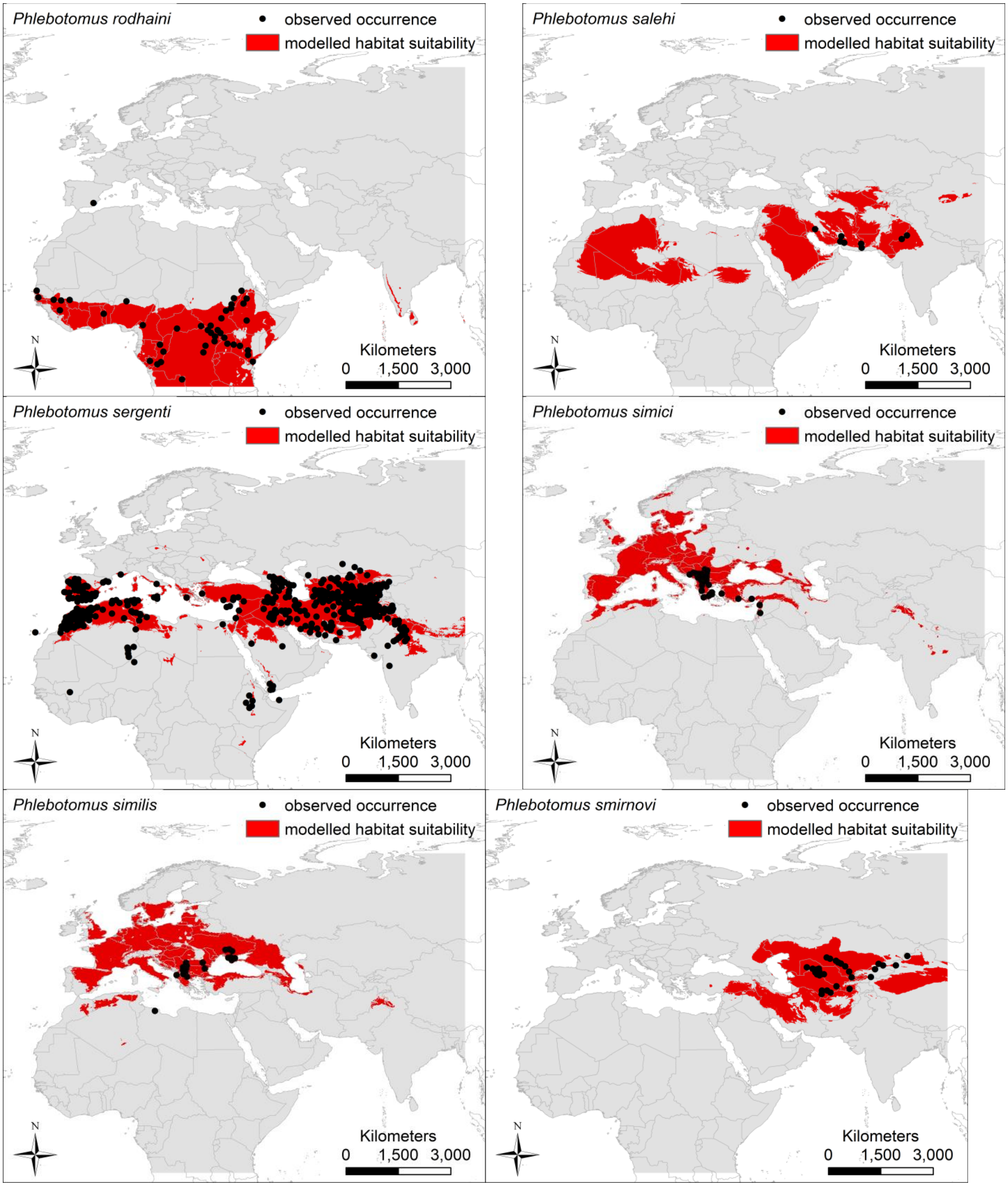
Phlebotomus rodhaini
observed occurrence
modelled habitat suitability
Kilometers
0 1,500 3,000
N
Phlebotomus salehi
observed occurrence
modelled habitat suitability
Kilometers
0 1,500 3,000
N
Phlebotomus sergenti
observed occurrence
modelled habitat suitability
Kilometers
0 1,500 3,000
N
Phlebotomus simici
observed occurrence
modelled habitat suitability
Kilometers
0 1,500 3,000
N
Phlebotomus similis
observed occurrence
modelled habitat suitability
Kilometers
0 1,500 3,000
N
Phlebotomus smirnovi
observed occurrence
modelled habitat suitability
Kilometers
0 1,500 3,000
N

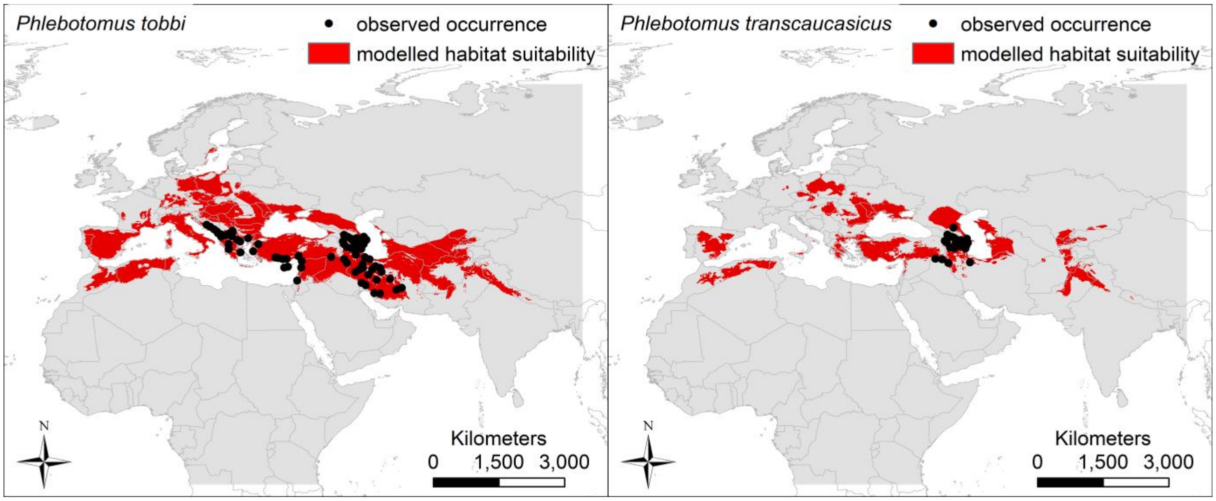

Fig. S1: Observed distribution and modelled habitat suitability for 32 *Phlebotomus* species known or suspected to be vector-competent for *Leishmania* parasites in Eurasia and Africa. The occurrence data for ecological niche modelling of the *Phlebotomus* species was mainly obtained from Artemiev und Neronov 1984 and supplemented by our own literature research (Akhoundi et al. 2012; Berdjane-Brouk et al. 2012; Boussaa et al. 2008; Giorgobiani et al. 2012; Benabdennbi et al. 1999; Rassi et al. 2009; Steinhauser 2005; Svobodová et al. 2009; Tabbabi et al. 2011; Tarallo et al. 2010; Weise 2004).

Table S1: Number of occurrence points for the 32 *Phlebotomus* species and AUC-values for each species.

**species**	**number of occurrence points used for modelling**	**AUC value**
P. alexandri	151	0.8989
P. argentipes	70	0.9580
P. ariasi	89	0.9833
P. balcanicus	49	0.9659
P. bergeroti	33	0.8841
P. caucasicus	185	0.9430
P. chabaudi	25	0.9884
P. chadlii	12	0.9786
P. duboscqi	54	0.9367
P. halepensis	47	0.9378
P. kandelakii	73	0.9600
P. langeroni	6	0.9488
P. longicuspis	60	0.9883
P. longiductus	80	0.9450
P. longipes	14	0.9930
P. martini	33	0.9780
P. mascittii	60	0.9836
P. mongolensis	96	0.9460
P. neglectus	90	0.9693
P. orientalis	30	0.9441

**species**	**number of occurrence points used for modelling**	**AUC value**
P. papatasi	319	0.8968
P. pedifer	6	0.9920
P. perfiliewi	142	0.9784
P. perniciosus	346	0.9626
P. rodhaini	49	0.9266
P. salehi	8	0.9280
P. sergenti	441	0.8946
P. simici	37	0.9690
P. similis	19	0.9691
P. smirnovi	29	0.9530
P. tobbi	79	0.9516
P. transcaucasicus	26	0.9770

Table S2: Variable importance of the eight climatic variables that were used for modelling. Shown are relative variable importance (Maxent permutation values) and rank-scaled variable importance with rank 1 for the most important variable and rank 8 for the least important variable. Abbreviations stand for: annual mean temperature (bio1), temperature seasonality (bio4), maximal temperature of the warmest month (bio05), minimal temperature of the coldest month (bio6), annual precipitation (bio12), precipitation of the wettest month (bio13), precipitation of the driest month (bio14) and precipitation seasonality (bio15).

	**Relative variable importance**								**Rank scaled variable importance**							
**species**	**bio1**	**bio4**	**bio5**	**bio6**	**bio12**	**bio13**	**bio14**	**bio15**	**bio1**	**bio4**	**bio5**	**bio6**	**bio12**	**bio13**	**bio14**	**bio15**
P. alexandri	18.6	22.1	26.7	10.7	6.9	1.1	8	5.9	3	2	1	4	6	8	5	7
P. argentipes	0.7	16.7	0	56	18.5	2.9	3.6	1.6	7	3	8	1	2	5	4	6
P. ariasi	25.2	28	5	4.6	9	0	4.5	23.9	2	1	5	6	4	8	7	3
P. balcanicus	12.4	13.8	8.9	47.7	3.3	3.3	10.4	0.3	3	2	5	1	6	6	4	8
P. bergeroti	0	57.8	1.2	4.9	16.6	0.1	16.4	3.1	8	1	6	4	2	7	3	5
P. caucasicus	25	24.9	5.1	12.5	5.8	5.6	9.5	11.6	1	2	8	3	6	7	5	4
P. chabaudi	21.5	24.1	26.5	19	0.8	3.8	2.9	1.5	3	2	1	4	8	5	6	7
P. chadlii	11.4	11.9	0.2	25.5	2.5	0.6	25.5	17.5	5	4	8	1	6	7	1	3
P. duboscqi	18.6	52.4	3.8	0	16.7	8.5	0	0	2	1	5	6	3	4	6	6
P. halepensis	7.6	8.7	13.8	45.4	10.9	11.2	0.3	2	6	5	2	1	4	3	8	7
P. kandelakii	25.1	28.7	6.5	11.3	10.3	12.6	2.8	2.6	2	1	6	4	5	3	7	8
P. langeroni	0	15.3	0	66	0	0	16.5	2.3	5	3	5	1	5	5	2	4
P. longicuspis	31.9	34.4	19.1	3.7	0	0.2	8	2.7	2	1	3	5	8	7	4	6
P. longiductus	18.7	8.8	6.2	27.5	8.9	9.4	13.5	7	2	6	8	1	5	4	3	7
P. longipes	0	73.1	0	26.7	0	0	0.2	0	4	1	4	2	4	4	3	4
P. martini	1.4	78.3	0.3	2.7	12.7	2.4	1	1.2	5	1	8	3	2	4	7	6
P. mascittii	24.5	12.9	3.8	33.9	5	0	1.9	18.1	2	4	6	1	5	8	7	3

P. mongolensis	31.9	10.7	4.8	16.8	10.9	0	23.2	1.6	1	5	6	3	4	8	2	7
species	bio1	bio4	bio5	bio6	bio12	bio13	bio14	bio15	bio1	bio4	bio5	bio6	bio12	bio13	bio14	bio15
P. neglectus	25.7	25	9	34.3	2.4	0.1	1.4	2.2	2	3	4	1	5	8	7	6
P. orientalis	0	78.6	1	4.9	14.9	0.6	0	0	6	1	4	3	2	5	6	6
P. papatasi	32.3	15.3	17.9	30.8	1.6	0.1	1.4	0.6	1	4	3	2	5	8	6	7
P. pedifer	0	84.3	0	4.1	5.5	0	4.7	1.4	6	1	6	4	2	6	3	5
P. perfiliewi	38.9	14.9	2.6	37.6	0.4	0	4.5	1	1	3	5	2	7	8	4	6
P. perniciosus	37.2	17.8	6.4	29.2	0.7	0	1.5	7.2	1	3	5	2	7	8	6	4
P. rodhaini	1.6	84.3	3.4	0	4.2	5.5	0.5	0.3	5	1	4	8	3	2	6	7
P. salehi	4.2	60.3	0	24.4	8.8	0	0	2.4	4	1	6	2	3	6	6	5
P. sergenti	32	17.1	11.7	10	2.8	6.9	11	8.4	1	2	3	5	8	7	4	6
P. simici	14.3	30	4.4	60.4	10.5	0.4	4.4	2.4	3	2	5	1	4	8	5	7
P. similis	0	7.5	1.9	63.7	3.8	0	19.6	3.5	7	3	6	1	4	7	2	5
P. smirnovi	19.3	20.6	9	34.4	0	1.6	15	0.1	3	2	5	1	8	6	4	7
P. tobbi	32.7	21.2	8.7	29.5	0.6	0.1	3	4.2	1	3	4	2	7	8	6	5
P. transcaucasicus	1.3	5.7	0	56.5	7.1	7.6	15.8	6	7	6	8	1	4	3	2	5
								**Average rank**	**3.47**	**2.50**	**5.09**	**2.69**	**4.81**	**6.03**	**4.72**	**5.69**

References

Akhoundi, Mohammad; Parvizi, Parviz; Baghaei, Ahmad; Depaquit, Jérôme (2012): The subgenus *Adlerius Nitzulescu* (Diptera, Psychodidae, Phlebotomus) in Iran. In: *Acta tropica* 122 (1), S. 7–15.

Artemiev, V. M.; Neronov, V. M. (Hg.) (1984): Distribution and ecology of sandflies of the Old World (genus *Phlebotomus*). Moscow: The USSR Committee for the Unesco Programme on Man and the Biosphere (MAB) Institute of Evolutionary Morphology and Animal Ecology; USSR Academy of Science.

Benabdennbi, I.; Pesson, B.; Cadi-Soussi, M.; Marquez, F. Morillas (1999): Morphological and isoenzymatic differentiation of sympatric populations of *Phlebotomus perniciosus* and *Phlebotomus longicuspis* (Diptera Psychodidae) in Northern Morocco. In: *Journal of Medical Entomology* 36 (1), S. 116–120. DOI: 10.1093/jmedent/36.1.116.

Berdjane-Brouk, Zohra; Charrel, Remi N.; Hamrioui, Boussad; Izri, Arezki (2012): First detection of *Leishmania infantum* DNA in *Phlebotomus longicuspis* Nitzulescu, 1930 from visceral leishmaniasis endemic focus in Algeria. In: *Parasitology research* 111 (1), S. 419–422. DOI: 10.1007/s00436-012-2858-1.

Boussaa, S.; Boumezzough, A.; Remy, P. E.; Glasser, N.; Pesson, B. (2008): Morphological and isoenzymatic differentiation of *Phlebotomus perniciosus* and *Phlebotomus longicuspis* (Diptera Psychodidae) in Southern Morocco. In: *Acta tropica* 106 (3), S. 184–189. DOI: 10.1016/j.actatropica.2008.03.011.

Giorgobiani, Ekaterina; Lawyer, Phillip G.; Babuadze, Giorgi; Dolidze, Nato; Jochim, Ryan C.; Tskhvaradze, Lamzira et al. (2012): Incrimination of *Phlebotomus kandelakii* and *Phlebotomus balcanicus* as vectors of *Leishmania infantum* in Tbilisi, Georgia. In: *PLoS neglected tropical diseases* 6 (4), S. e1609. DOI: 10.1371/journal.pntd.0001609.

Rassi, Y.; Javadian, E.; Nadim, A.; Rafizadeh, S.; Zahraii, A.; Azizi, K.; Mohebali, M. (2009): *Phlebotomus perfiliewi transcaucasicus*, a Vector of *Leishmania infantum* in Northwestern Iran. In: *J Med Entomol* 46 (5), S. 1094–1098. DOI: 10.1603/033.046.0516.

Steinhauser, Irmgard (2005): Untersuchung zur Verbreitung von Sandmücken (Phlebotomen) in Deutschland mit Hilfe geographischer Informationssysteme (GIS). Diploma thesis. Friedrich-Wilhelm-Universität, Bonn. Institut für Medizinische Parasitologie. Online available http://parasitosen.de/images/downloads/publikationen/diplomarbeit_irmgard_steinhausen.pdf, Accessed 08.05.2017.

Svobodová, Milena; Alten, Bulent; Zídková, Lenka; Dvořák, Vít; Hlavačková, Jitka; Myšková, Jitka et al. (2009): Cutaneous leishmaniasis caused by *Leishmania infantum* transmitted by *Phlebotomus tobbi*. In: *International journal for parasitology* 39 (2), S. 251–256.

Tabbabi, Ahmed; Bousslimi, Nadia; Rhim, Adel; Aoun, Karim; Bouratbine, Aida (2011): First report on natural infection of *Phlebotomus sergenti* with *Leishmania* promastigotes in the cutaneous leishmaniasis focus in southeastern Tunisia. In: *The American journal of tropical medicine and hygiene* 85 (4), S. 646–647. DOI: 10.4269/ajtmh.2011.10-0681.

Tarallo, Viviana D.; Dantas-Torres, Filipe; Lia, Riccardo P.; Otranto, Domenico (2010): Phlebotomine sand fly population dynamics in a leishmaniasis endemic peri-urban area in southern Italy. In: *Acta tropica* 116 (3), S. 227–234.

Weise, Miriam (2004): Reisetiermedizinisch und epidemiologisch wichtige Arten der kaninen Parasitenfauna in europäischen Anrainerstaaten des Mittelmeeres und in Portugal für Hunde in Deutschland. Dissertation. Ludwig-Maximilian-Universität, München. Institut für Vergleichende Tropenmedizin und Parasitologie. Online available https://edoc.ub.uni-muenchen.de/1876/1/Weise_Miriam.pdf, Accessed 08.05.2017.
