# Supplementary material for: Leishmaniasis in Eurasia and Africa: geographical distribution of vector species and pathogens
Source: R Soc Open Sci. 2019 May 22;6(5):190334. doi: 10.1098/rsos.190334 (PMC6549972; doi:10.1098/rsos.190334)
Supplement: Suppelentary material part II [file rsos190334supp2.pdf]

Table S3: Occurrence records of the *Phlebotomus* species used for ecological niche modelling. The occurrence data was mainly obtained from [1] and supplemented by our own literature research [2–12].

**species**	**lon**	**lat**
Phlebotomus alexandri	18.2464685	42.5117238
Phlebotomus alexandri	24.5661124	46.7515566
Phlebotomus alexandri	31.1978521	29.8988298
Phlebotomus alexandri	18.2137852	42.2732218
Phlebotomus alexandri	18.0021215	43.1341365
Phlebotomus alexandri	3.974047028	24.98110564
Phlebotomus alexandri	5.818129307	25.04059217
Phlebotomus alexandri	-8.785812616	29.05593262
Phlebotomus alexandri	-7.893514739	30.03746028
Phlebotomus alexandri	-6.52532466	30.09694681
Phlebotomus alexandri	-5.662770046	31.88154256
Phlebotomus alexandri	-3.907917554	32.03025887
Phlebotomus alexandri	-1.706916124	32.29794824
Phlebotomus alexandri	0.851004458	34.52869293
Phlebotomus alexandri	4.717628592	34.2312603
Phlebotomus alexandri	3.408925039	32.5656376
Phlebotomus alexandri	8.465279676	34.61792272
Phlebotomus alexandri	9.208861241	33.63639505
Phlebotomus alexandri	9.77398323	32.62512412
Phlebotomus alexandri	6.109613281	34.55248754
Phlebotomus alexandri	6.442737821	35.50427194
Phlebotomus alexandri	-1.4573	37.403633
Phlebotomus alexandri	33.72920691	5.296221318
Phlebotomus alexandri	36.584754	14.468884
Phlebotomus alexandri	41.7598878	12.16691497
Phlebotomus alexandri	42.56295589	11.9884554
Phlebotomus alexandri	44.13934881	15.17098449
Phlebotomus alexandri	45.35882257	14.69509229
Phlebotomus alexandri	48.27564519	24.22483362
Phlebotomus alexandri	48.08528831	25.74768866
Phlebotomus alexandri	69.01264785	26.7708569
Phlebotomus alexandri	63.03425208	26.41393775
Phlebotomus alexandri	64.07526627	28.16879024
Phlebotomus alexandri	62.40964356	30.96465692
Phlebotomus alexandri	59.55429036	29.1503179
Phlebotomus alexandri	57.7696946	27.87135761
Phlebotomus alexandri	56.342018	29.35852074
Phlebotomus alexandri	58.03738396	30.54825124
Phlebotomus alexandri	55.36049033	31.11337323
Phlebotomus alexandri	56.25278821	32.30310373
Phlebotomus alexandri	53.21897543	28.79339875
Phlebotomus alexandri	54.76562508	29.68569663
Phlebotomus alexandri	53.18923217	30.1021023

species	lon	lat
Phlebotomus alexandri	52.2077045	31.11337323
Phlebotomus alexandri	50.83951442	31.55952217
Phlebotomus alexandri	51.22617684	32.54104984
Phlebotomus alexandri	52.35642081	33.90923991
Phlebotomus alexandri	50.86925768	35.4261463
Phlebotomus alexandri	48.22210732	34.41487538
Phlebotomus alexandri	49.94721654	32.92771225
Phlebotomus alexandri	48.46005342	32.51130657
Phlebotomus alexandri	48.1257	30.813467
Phlebotomus alexandri	49.85798676	30.81594061
Phlebotomus alexandri	50.7800279	30.16158883
Phlebotomus alexandri	43.40369878	32.89796899
Phlebotomus alexandri	44.68265907	32.00567111
Phlebotomus alexandri	46.43751156	32.24361721
Phlebotomus alexandri	43.07652289	36.97279596
Phlebotomus alexandri	43.07652289	35.72357893
Phlebotomus alexandri	45.75341652	36.52664702
Phlebotomus alexandri	46.0776	35.192233
Phlebotomus alexandri	44.47445623	36.16972787
Phlebotomus alexandri	44.74214559	35.15845694
Phlebotomus alexandri	44.14728034	34.05795623
Phlebotomus alexandri	44.89086191	34.15908332
Phlebotomus alexandri	45.55711099	33.01694204
Phlebotomus alexandri	55.796633	24.76575
Phlebotomus alexandri	58.01061503	34.66412392
Phlebotomus alexandri	59.37880511	35.02104307
Phlebotomus alexandri	60.36033277	36.30000336
Phlebotomus alexandri	56.01781644	39.6015055
Phlebotomus alexandri	55.9319	38.165833
Phlebotomus alexandri	56.93985758	37.57896365
Phlebotomus alexandri	57.56446609	38.47126153
Phlebotomus alexandri	57.68343914	39.77996508
Phlebotomus alexandri	59.23008879	40.40457359
Phlebotomus alexandri	59.11111574	38.67946437
Phlebotomus alexandri	59.97367036	38.08459911
Phlebotomus alexandri	61.1336576	37.4599906
Phlebotomus alexandri	62.76953704	37.66819344
Phlebotomus alexandri	62.38287463	36.59743598
Phlebotomus alexandri	63.89978102	37.16255797
Phlebotomus alexandri	63.66183492	35.46719201
Phlebotomus alexandri	65.29771436	36.80563882
Phlebotomus alexandri	64.6488	36.461533
Phlebotomus alexandri	64.94079521	34.75335371
Phlebotomus alexandri	65.17874131	35.46719201
Phlebotomus alexandri	64.94079521	33.5636232
Phlebotomus alexandri	65.9818094	34.2774615

species	lon	lat
Phlebotomus alexandri	66.51718812	35.49693527
Phlebotomus alexandri	64.97053847	32.43337922
Phlebotomus alexandri	65.77360656	31.68979766
Phlebotomus alexandri	67.32025621	32.22517639
Phlebotomus alexandri	66.80945	31.1759
Phlebotomus alexandri	65.9818094	30.14314801
Phlebotomus alexandri	68.98587892	28.44778204
Phlebotomus alexandri	70.53252857	29.04264729
Phlebotomus alexandri	71.98994844	29.69699907
Phlebotomus alexandri	71.3058534	30.70827
Phlebotomus alexandri	71.72225907	31.80877071
Phlebotomus alexandri	70.77047467	32.64158206
Phlebotomus alexandri	69.43202786	31.21390546
Phlebotomus alexandri	68.3484	31.776033
Phlebotomus alexandri	68.86690587	32.52260901
Phlebotomus alexandri	70.15165	33.468267
Phlebotomus alexandri	70.71740267	33.98628348
Phlebotomus alexandri	72.04943496	33.83131257
Phlebotomus alexandri	71.98994844	34.87232676
Phlebotomus alexandri	68.03409452	34.18823172
Phlebotomus alexandri	68.27204062	35.28873243
Phlebotomus alexandri	69.49151438	35.55642179
Phlebotomus alexandri	70.47304205	34.69386718
Phlebotomus alexandri	74.87563977	33.75992874
Phlebotomus alexandri	63.91822184	41.61215005
Phlebotomus alexandri	66.01175	42.196233
Phlebotomus alexandri	66.23819632	45.28841731
Phlebotomus alexandri	61.41978778	42.71859942
Phlebotomus alexandri	62.0281	40.8235
Phlebotomus alexandri	63.0734	39.6627
Phlebotomus alexandri	64.4131	39.0221
Phlebotomus alexandri	65.98835292	39.04233217
Phlebotomus alexandri	67.16618611	40.07739771
Phlebotomus alexandri	65.52435802	40.79123601
Phlebotomus alexandri	68.20125165	42.93275091
Phlebotomus alexandri	69.4402	41.467667
Phlebotomus alexandri	69.93725	40.205233
Phlebotomus alexandri	67.63018101	38.54264536
Phlebotomus alexandri	71.83295	39.327833
Phlebotomus alexandri	68.3083274	37.54327173
Phlebotomus alexandri	69.62892825	37.97157472
Phlebotomus alexandri	68.51915	39.5368
Phlebotomus alexandri	69.37908485	38.79248876
Phlebotomus alexandri	73.01966019	41.39799856
Phlebotomus alexandri	74.12610956	41.07677133
Phlebotomus alexandri	75.23255892	41.75491771

species	lon	lat
Phlebotomus alexandri	66.5262	37.6999
Phlebotomus alexandri	66.64270469	36.54984676
Phlebotomus alexandri	67.71346214	36.5201035
Phlebotomus alexandri	69.17088201	36.5201035
Phlebotomus alexandri	70.06317989	36.93650918
Phlebotomus alexandri	70.33086925	39.13751061
Phlebotomus alexandri	60.79315	41.279
Phlebotomus alexandri	46.56481272	38.58428592
Phlebotomus alexandri	47.8319	39.771433
Phlebotomus alexandri	45.34815	39.140567
Phlebotomus alexandri	80.6803349	46.97188597
Phlebotomus alexandri	34.16187223	37.52542578
Phlebotomus alexandri	35.137395	31.868774
Phlebotomus alexandri	92.64664429	43.64302002
Phlebotomus alexandri	96.64413878	44.42824216
Phlebotomus alexandri	100.4274818	39.93106085
Phlebotomus argentipes	73.33960291	31.78274391
Phlebotomus argentipes	74.3146	30.786467
Phlebotomus argentipes	77.38468662	31.54479781
Phlebotomus argentipes	77.20622704	29.46276943
Phlebotomus argentipes	77.74160577	28.4514985
Phlebotomus argentipes	76.78982136	27.14279495
Phlebotomus argentipes	75.65957739	26.01255097
Phlebotomus argentipes	74.94573909	25.47717224
Phlebotomus argentipes	72.98268376	24.34692826
Phlebotomus argentipes	73.51806248	21.84849421
Phlebotomus argentipes	74.17241426	18.39827575
Phlebotomus argentipes	74.70779298	17.38700482
Phlebotomus argentipes	73.02595	18.8813
Phlebotomus argentipes	73.19295	17.611267
Phlebotomus argentipes	73.99395468	16.79213957
Phlebotomus argentipes	77.86057882	13.16346154
Phlebotomus argentipes	77.26571357	9.534783503
Phlebotomus argentipes	79.34774195	13.52038069
Phlebotomus argentipes	77.45607045	8.886380379
Phlebotomus argentipes	75.78330936	19.91042322
Phlebotomus argentipes	76.8302722	19.91042322
Phlebotomus argentipes	76.8302722	19.91042322
Phlebotomus argentipes	76.29251401	19.12996001
Phlebotomus argentipes	78.96940765	17.55951575
Phlebotomus argentipes	81.14661447	18.45181362
Phlebotomus argentipes	81.71768511	17.48813192
Phlebotomus argentipes	84.35888682	19.87949023
Phlebotomus argentipes	79.04079148	21.20009108
Phlebotomus argentipes	76.720817	21.41424257
Phlebotomus argentipes	79.43340254	30.08737794

species	lon	lat
Phlebotomus argentipes	79.54047829	28.3384741
Phlebotomus argentipes	80.2900085	26.73233792
Phlebotomus argentipes	81.28938213	25.30466132
Phlebotomus argentipes	82.36013958	24.87635834
Phlebotomus argentipes	81.68199319	23.87698472
Phlebotomus argentipes	84.10904342	25.94711579
Phlebotomus argentipes	83.78781618	24.87635834
Phlebotomus argentipes	84.75149789	24.69789876
Phlebotomus argentipes	85.82225534	24.98343408
Phlebotomus argentipes	87.24993195	25.23327749
Phlebotomus argentipes	88.17285	24.572867
Phlebotomus argentipes	89.07021962	25.44742898
Phlebotomus argentipes	90.9261992	25.91142388
Phlebotomus argentipes	93.03202219	25.94711579
Phlebotomus argentipes	90.4622043	24.59082302
Phlebotomus argentipes	89.32006302	24.01975238
Phlebotomus argentipes	86.82162897	23.69852514
Phlebotomus argentipes	85.7151796	23.66283323
Phlebotomus argentipes	84.5373464	23.52006556
Phlebotomus argentipes	83.25243746	23.30591407
Phlebotomus argentipes	83.25243746	22.55638386
Phlebotomus argentipes	84.78718981	21.73546981
Phlebotomus argentipes	86.14348258	21.6997779
Phlebotomus argentipes	87.96377025	22.84191918
Phlebotomus argentipes	88.8661	22.766233
Phlebotomus argentipes	80.43277616	7.922698672
Phlebotomus argentipes	80.46846808	7.101784625
Phlebotomus argentipes	81.18230638	6.959016964
Phlebotomus argentipes	96.1372188	17.48813192
Phlebotomus argentipes	99.38518308	19.09426809
Phlebotomus argentipes	103.1685261	19.77241448
Phlebotomus argentipes	100.0990214	14.91831403
Phlebotomus argentipes	101.8836171	14.3829353
Phlebotomus argentipes	108.5223133	14.56139488
Phlebotomus argentipes	108.4509295	12.81249104
Phlebotomus argentipes	102.1632038	5.22201043
Phlebotomus argentipes	101.5683385	3.496901201
Phlebotomus argentipes	103.3659	4.129033
Phlebotomus argentipes	116.78665	4.383867
Phlebotomus argentipes	117.897241	5.276291884
Phlebotomus ariasi	-7.357375302	41.53653888
Phlebotomus ariasi	-7.262196862	40.85125411
Phlebotomus ariasi	-6.92155	40.2639
Phlebotomus ariasi	-5.663199066	40.16596934
Phlebotomus ariasi	-6.139091267	40.85125411
Phlebotomus ariasi	-6.062948515	41.4984675

species	lon	lat
Phlebotomus ariasi	-5.015985672	41.06064668
Phlebotomus ariasi	-4.673343287	40.31825484
Phlebotomus ariasi	-4.083236958	41.36521769
Phlebotomus ariasi	-3.96902283	40.71800429
Phlebotomus ariasi	-3.816737325	40.18500503
Phlebotomus ariasi	-2.350989346	40.45150466
Phlebotomus ariasi	-2.998202739	40.83221842
Phlebotomus ariasi	-2.941095675	41.47943181
Phlebotomus ariasi	-2.941095675	42.08857383
Phlebotomus ariasi	0.85533436	47.7778651
Phlebotomus ariasi	1.493029909	46.79752716
Phlebotomus ariasi	0.826780828	46.01706395
Phlebotomus ariasi	-0.001271603	44.83685129
Phlebotomus ariasi	0.065353306	43.66615648
Phlebotomus ariasi	1.359780093	44.83685129
Phlebotomus ariasi	1.283637341	44.2752985
Phlebotomus ariasi	0.731602387	43.13315721
Phlebotomus ariasi	1.569172661	43.24737134
Phlebotomus ariasi	1.87374367	41.39139176
Phlebotomus ariasi	2.520957064	41.83873043
Phlebotomus ariasi	2.930224357	42.19089065
Phlebotomus ariasi	2.482885688	42.16233712
Phlebotomus ariasi	2.064100551	42.18137281
Phlebotomus ariasi	1.566151084	42.41399846
Phlebotomus ariasi	2.235421743	42.88569327
Phlebotomus ariasi	3.028	42.861233
Phlebotomus ariasi	2.806492384	43.53290666
Phlebotomus ariasi	3.653580502	43.54242451
Phlebotomus ariasi	4.40575	43.388567
Phlebotomus ariasi	5.105051716	43.47104068
Phlebotomus ariasi	5.94262199	43.52814774
Phlebotomus ariasi	6.81826364	43.64236187
Phlebotomus ariasi	7.267676	43.79669
Phlebotomus ariasi	8.1632	43.9428
Phlebotomus ariasi	5.688019662	43.24499188
Phlebotomus ariasi	5.961657678	43.22119727
Phlebotomus ariasi	6.330474134	43.25688918
Phlebotomus ariasi	7.853329178	44.26816011
Phlebotomus ariasi	8.257837549	44.43472238
Phlebotomus ariasi	8.983573156	44.50610621
Phlebotomus ariasi	4.997975971	45.06527955
Phlebotomus ariasi	5.842684628	44.89871728
Phlebotomus ariasi	6.473241794	44.91061458
Phlebotomus ariasi	7.329847756	44.9582038
Phlebotomus ariasi	5.813337942	44.15057508
Phlebotomus ariasi	6.336819363	43.67468288

species	lon	lat
Phlebotomus ariasi	3.648028427	44.07919125
Phlebotomus ariasi	4.433250559	44.05539664
Phlebotomus ariasi	3.005573955	44.12678047
Phlebotomus ariasi	5.07570503	44.10298586
Phlebotomus ariasi	6.83815	44.351267
Phlebotomus ariasi	7.478960646	44.35282927
Phlebotomus ariasi	4.03444298	44.89143018
Phlebotomus ariasi	4.557924401	44.65348408
Phlebotomus ariasi	3.177837018	44.84384096
Phlebotomus ariasi	5.927999	43.124228
Phlebotomus ariasi	6.76837	43.42519
Phlebotomus ariasi	7.2619532	43.7101728
Phlebotomus ariasi	5.36978	43.296482
Phlebotomus ariasi	3.2583626	43.5912356
Phlebotomus ariasi	3.6673942	43.9755057
Phlebotomus ariasi	-1.1306544	37.9922399
Phlebotomus ariasi	-1.1306544	37.9922399
Phlebotomus ariasi	-2.302446	37.1035929
Phlebotomus ariasi	-3.59855709	37.1773363
Phlebotomus ariasi	-4.11044449	36.840268
Phlebotomus ariasi	-5.6635397	40.9701039
Phlebotomus ariasi	-3.7037902	40.4167754
Phlebotomus ariasi	0.810652	41.2133495
Phlebotomus ariasi	0.8454749	41.2344554
Phlebotomus ariasi	2.1734035	41.3850639
Phlebotomus ariasi	1.5208624	41.5911589
Phlebotomus ariasi	-1.6760691	42.6953909
Phlebotomus ariasi	-7.78848089	41.1655901
Phlebotomus ariasi	-7.747452	41.299773
Phlebotomus ariasi	-7.909307399	38.5707119
Phlebotomus ariasi	-9.139336599	38.7222524
Phlebotomus ariasi	-8.6406106	41.1514848
Phlebotomus ariasi	-7.930834	37.0179538
Phlebotomus ariasi	-8.5335055	37.1417854
Phlebotomus ariasi	4.2040933	36.63729932
Phlebotomus ariasi	-9.6035176	30.4751364
Phlebotomus ariasi	-5.661571	32.9340471
Phlebotomus balcanicus	19.10765	44.475967
Phlebotomus balcanicus	19.52745	44.018867
Phlebotomus balcanicus	20.28539927	44.33074665
Phlebotomus balcanicus	20.99923757	44.33074665
Phlebotomus balcanicus	21.2497	45.280367
Phlebotomus balcanicus	21.6119	44.728333
Phlebotomus balcanicus	21.86179218	44.2117736
Phlebotomus balcanicus	22.4645	44.7176
Phlebotomus balcanicus	20.70180494	43.34921899

species	lon	lat
Phlebotomus balcanicus	21.38589998	43.31947573
Phlebotomus balcanicus	22.04025176	43.28973246
Phlebotomus balcanicus	21.523849	42.377276
Phlebotomus balcanicus	21.56435956	42.69486721
Phlebotomus balcanicus	22.44625	42.735667
Phlebotomus balcanicus	20.88026452	42.45692111
Phlebotomus balcanicus	22.360773	42.369484
Phlebotomus balcanicus	20.6265	41.970033
Phlebotomus balcanicus	22.5478	41.189067
Phlebotomus balcanicus	24.50894255	46.02611262
Phlebotomus balcanicus	24.33048297	44.27126013
Phlebotomus balcanicus	25.87713263	44.47946297
Phlebotomus balcanicus	27.86993122	44.41997644
Phlebotomus balcanicus	28.40530995	45.01484169
Phlebotomus balcanicus	28.4472	45.642067
Phlebotomus balcanicus	28.55402626	46.2045722
Phlebotomus balcanicus	30.9952	46.651433
Phlebotomus balcanicus	32.86679933	46.56149135
Phlebotomus balcanicus	33.4374	45.2689
Phlebotomus balcanicus	33.848327	44.92561191
Phlebotomus balcanicus	34.4872	44.8321
Phlebotomus balcanicus	34.94882771	45.37176084
Phlebotomus balcanicus	39.48467526	45.07432822
Phlebotomus balcanicus	43.88667812	43.9738275
Phlebotomus balcanicus	45.04666536	43.11127289
Phlebotomus balcanicus	46.17690933	42.93281331
Phlebotomus balcanicus	44.36257032	42.27846154
Phlebotomus balcanicus	45.10615188	42.0702587
Phlebotomus balcanicus	46.1068	42.006767
Phlebotomus balcanicus	44.80871926	40.76155515
Phlebotomus balcanicus	47.0848	39.340433
Phlebotomus balcanicus	48.8065	38.318567
Phlebotomus balcanicus	44.7568846	41.7095991
Phlebotomus balcanicus	44.7826472	41.6935204
Phlebotomus balcanicus	48.082675	39.0393593
Phlebotomus balcanicus	57.6789735	36.2148413
Phlebotomus balcanicus	47.5495505	38.0058809
Phlebotomus balcanicus	47.1338639	38.0841098
Phlebotomus balcanicus	46.3210443	38.0679372
Phlebotomus balcanicus	44.5701991	34.8566834
Phlebotomus bergeroti	-5.439653031	33.3391195
Phlebotomus bergeroti	-7.319427225	30.57894473
Phlebotomus bergeroti	-6.177285943	30.84068545
Phlebotomus bergeroti	-5.24929615	31.34037226
Phlebotomus bergeroti	-4.583047069	32.17318361
Phlebotomus bergeroti	-6.581794314	29.60336572

species	lon	lat
Phlebotomus bergeroti	9.431978256	25.5225901
Phlebotomus bergeroti	4.97048887	21.50724965
Phlebotomus bergeroti	6.576625049	21.03135745
Phlebotomus bergeroti	7.885328602	17.90831488
Phlebotomus bergeroti	-13.20264456	21.20981702
Phlebotomus bergeroti	-0.323811868	12.82221698
Phlebotomus bergeroti	16.70123163	21.96926166
Phlebotomus bergeroti	17.23661035	20.48209853
Phlebotomus bergeroti	18.27762454	19.44108434
Phlebotomus bergeroti	20.12170682	14.17652687
Phlebotomus bergeroti	34.80892988	17.14490447
Phlebotomus bergeroti	35.55251145	18.3941215
Phlebotomus bergeroti	36.29609301	16.9664449
Phlebotomus bergeroti	37.69402635	16.81772858
Phlebotomus bergeroti	41.20373134	9.441399466
Phlebotomus bergeroti	42.12577248	10.18498103
Phlebotomus bergeroti	42.92884057	10.80958954
Phlebotomus bergeroti	46.5575186	9.411656204
Phlebotomus bergeroti	44.26728738	14.55724063
Phlebotomus bergeroti	44.02934128	19.91102789
Phlebotomus bergeroti	47.24161364	14.70595694
Phlebotomus bergeroti	48.10416825	15.98491723
Phlebotomus bergeroti	41.02527176	21.66588038
Phlebotomus bergeroti	44.62420653	25.80019388
Phlebotomus bergeroti	43.30162279	14.1188646
Phlebotomus bergeroti	44.8815849	13.56682964
Phlebotomus bergeroti	55.72835789	24.53614488
Phlebotomus caucasicus	43.48629076	42.99221381
Phlebotomus caucasicus	44.2256	42.728933
Phlebotomus caucasicus	44.61415528	42.492527
Phlebotomus caucasicus	45.12811886	42.32120581
Phlebotomus caucasicus	45.06465	42.7824
Phlebotomus caucasicus	45.6991895	41.57881398
Phlebotomus caucasicus	44.12874524	40.62227065
Phlebotomus caucasicus	44.72836941	40.59371712
Phlebotomus caucasicus	45.27088652	40.49377976
Phlebotomus caucasicus	45.5689	40.065833
Phlebotomus caucasicus	46.18459954	39.97981618
Phlebotomus caucasicus	45.0419	39.754667
Phlebotomus caucasicus	45.48045	39.537867
Phlebotomus caucasicus	46.07038542	39.45157584
Phlebotomus caucasicus	44.45711085	40.15113737
Phlebotomus caucasicus	44.95679767	38.63780017
Phlebotomus caucasicus	45.88478746	39.09465669
Phlebotomus caucasicus	46.5087	39.063767
Phlebotomus caucasicus	46.91271461	38.43792545

species	lon	lat
Phlebotomus caucasicus	46.98409844	39.68000409
Phlebotomus caucasicus	47.04120551	40.15113737
Phlebotomus caucasicus	47.44095496	40.53661006
Phlebotomus caucasicus	46.86988431	40.76503831
Phlebotomus caucasicus	48.65448007	41.32183219
Phlebotomus caucasicus	48.96856892	40.86497568
Phlebotomus caucasicus	49.19699718	40.43667269
Phlebotomus caucasicus	48.36894475	40.72220802
Phlebotomus caucasicus	48.06913266	40.33673533
Phlebotomus caucasicus	47.66938321	39.97981618
Phlebotomus caucasicus	48.36894475	40.17969091
Phlebotomus caucasicus	48.85435479	39.83704852
Phlebotomus caucasicus	48.79724773	39.35163848
Phlebotomus caucasicus	47.82642764	38.29515779
Phlebotomus caucasicus	45.09956533	37.78119421
Phlebotomus caucasicus	46.61290253	37.2815074
Phlebotomus caucasicus	47.34101759	35.04005513
Phlebotomus caucasicus	46.85560755	34.08351181
Phlebotomus caucasicus	48.82580126	33.91219062
Phlebotomus caucasicus	49.49680926	33.11269172
Phlebotomus caucasicus	50.35341523	35.52546518
Phlebotomus caucasicus	51.45272621	35.53974194
Phlebotomus caucasicus	52.62342103	34.81162688
Phlebotomus caucasicus	52.20939481	33.64093206
Phlebotomus caucasicus	53.24017732	35.36842075
Phlebotomus caucasicus	54.41087213	35.88238433
Phlebotomus caucasicus	55.31030839	36.4820085
Phlebotomus caucasicus	49.80233206	40.50520117
Phlebotomus caucasicus	52.68544564	29.55155072
Phlebotomus caucasicus	54.14286551	28.57002305
Phlebotomus caucasicus	54.82696055	31.8715252
Phlebotomus caucasicus	58.99101731	31.6335791
Phlebotomus caucasicus	60.00228824	32.02024151
Phlebotomus caucasicus	60.18074781	32.73407982
Phlebotomus caucasicus	53.13159458	43.29293803
Phlebotomus caucasicus	57.80128681	45.01804726
Phlebotomus caucasicus	57.93042214	42.70203855
Phlebotomus caucasicus	57.87093561	41.8811245
Phlebotomus caucasicus	57.46642724	41.32195116
Phlebotomus caucasicus	57.93042214	41.00072393
Phlebotomus caucasicus	55.90788028	40.83416166
Phlebotomus caucasicus	56.57412936	40.45344789
Phlebotomus caucasicus	55.84839376	40.2392964
Phlebotomus caucasicus	54.87281474	39.88237725
Phlebotomus caucasicus	54.09948992	39.68012307
Phlebotomus caucasicus	54.90850666	39.33510122

species	lon	lat
Phlebotomus caucasicus	54.82522552	38.06208958
Phlebotomus caucasicus	56.31238865	38.85920902
Phlebotomus caucasicus	55.89598298	39.44217697
Phlebotomus caucasicus	56.47895092	37.95501384
Phlebotomus caucasicus	57.98990866	39.31130661
Phlebotomus caucasicus	57.46642724	39.94186378
Phlebotomus caucasicus	56.01495603	37.1578944
Phlebotomus caucasicus	57.15709731	37.08651057
Phlebotomus caucasicus	58.40631434	36.64631029
Phlebotomus caucasicus	58.38251973	38.68074945
Phlebotomus caucasicus	59.47707179	38.87110633
Phlebotomus caucasicus	59.89347747	40.77467513
Phlebotomus caucasicus	60.01601971	37.28876476
Phlebotomus caucasicus	60.2301712	38.3357276
Phlebotomus caucasicus	61.11057177	38.35952221
Phlebotomus caucasicus	61.77682085	38.41900873
Phlebotomus caucasicus	61.74112894	38.84731172
Phlebotomus caucasicus	61.24144213	37.89552731
Phlebotomus caucasicus	61.56266936	37.47912164
Phlebotomus caucasicus	61.90769121	37.12220249
Phlebotomus caucasicus	62.06235617	36.69389951
Phlebotomus caucasicus	62.71670795	36.40836418
Phlebotomus caucasicus	62.91896214	36.96753752
Phlebotomus caucasicus	62.72860526	37.41963511
Phlebotomus caucasicus	62.90706483	38.13347341
Phlebotomus caucasicus	62.10994539	37.88363001
Phlebotomus caucasicus	60.58946981	42.53547627
Phlebotomus caucasicus	61.19623237	43.38018493
Phlebotomus caucasicus	63.26636344	44.32007203
Phlebotomus caucasicus	62.07663294	41.72645953
Phlebotomus caucasicus	63.25446614	42.38081131
Phlebotomus caucasicus	62.90944429	40.95313471
Phlebotomus caucasicus	64.45609395	41.78594606
Phlebotomus caucasicus	65.89566785	42.76152507
Phlebotomus caucasicus	65.24131608	41.15538889
Phlebotomus caucasicus	64.32522359	39.91806917
Phlebotomus caucasicus	63.4191	39.4383
Phlebotomus caucasicus	64.07645	39.038767
Phlebotomus caucasicus	63.52810415	38.39521412
Phlebotomus caucasicus	63.57569337	37.8717327
Phlebotomus caucasicus	63.59948798	37.21738093
Phlebotomus caucasicus	63.83743408	36.72959142
Phlebotomus caucasicus	63.3972338	36.14662347
Phlebotomus caucasicus	63.73035834	35.59934744
Phlebotomus caucasicus	64.61075891	36.07523964
Phlebotomus caucasicus	65.58633792	36.52733724

species	lon	lat
Phlebotomus caucasicus	64.30142898	37.30066206
Phlebotomus caucasicus	65.0861	37.237767
Phlebotomus caucasicus	64.8249104	37.90742462
Phlebotomus caucasicus	64.65834813	38.53798178
Phlebotomus caucasicus	66.25258701	37.12220249
Phlebotomus caucasicus	65.87544244	37.88363001
Phlebotomus caucasicus	65.6368	38.343533
Phlebotomus caucasicus	65.81595591	39.90617186
Phlebotomus caucasicus	67.55296244	44.93873189
Phlebotomus caucasicus	69.00443366	43.60623373
Phlebotomus caucasicus	66.75584301	40.31068023
Phlebotomus caucasicus	67.29122173	39.92996647
Phlebotomus caucasicus	66.99378911	39.39458775
Phlebotomus caucasicus	66.10149123	39.44217697
Phlebotomus caucasicus	66.57738343	38.91869555
Phlebotomus caucasicus	67.14845407	38.70454406
Phlebotomus caucasicus	67.26742712	38.19295994
Phlebotomus caucasicus	67.27932443	37.50291625
Phlebotomus caucasicus	68.24300614	37.24117554
Phlebotomus caucasicus	68.0568	38.4934
Phlebotomus caucasicus	67.8133	39.108367
Phlebotomus caucasicus	68.98063905	38.97818207
Phlebotomus caucasicus	68.61182259	38.12157611
Phlebotomus caucasicus	68.59992529	40.48913981
Phlebotomus caucasicus	69.59929891	41.98820024
Phlebotomus caucasicus	69.4491	37.224433
Phlebotomus caucasicus	69.90030073	37.72896504
Phlebotomus caucasicus	70.60224172	38.08588419
Phlebotomus caucasicus	70.04306839	38.58557101
Phlebotomus caucasicus	70.26911718	39.19233356
Phlebotomus caucasicus	70.04306839	40.37016676
Phlebotomus caucasicus	70.16204144	41.26246464
Phlebotomus caucasicus	71.43895	41.205633
Phlebotomus caucasicus	70.20011281	42.51168166
Phlebotomus caucasicus	72.19886006	42.19759281
Phlebotomus caucasicus	71.37080763	44.33910772
Phlebotomus caucasicus	72.96980542	45.03866925
Phlebotomus caucasicus	72.7699307	41.29815655
Phlebotomus caucasicus	74.26899113	41.56941511
Phlebotomus caucasicus	71.65634295	38.92821339
Phlebotomus caucasicus	72.12747623	38.48563364
Phlebotomus caucasicus	71.85621767	37.9573933
Phlebotomus caucasicus	70.89967435	37.74324181
Phlebotomus caucasicus	70.31432694	37.31493883
Phlebotomus caucasicus	70.21438958	36.78669848
Phlebotomus caucasicus	70.38571077	35.70166427

species	lon	lat
Phlebotomus caucasicus	72.37018125	36.37267227
Phlebotomus caucasicus	71.3202	36.242167
Phlebotomus caucasicus	70.82829052	35.24480775
Phlebotomus caucasicus	71.84194091	35.28763805
Phlebotomus caucasicus	71.4877	35.833933
Phlebotomus caucasicus	67.7540269	32.95100734
Phlebotomus caucasicus	69.4748531	34.21116989
Phlebotomus caucasicus	71.4799	37.5011
Phlebotomus caucasicus	74.97894298	38.78945116
Phlebotomus caucasicus	74.92183592	39.11305785
Phlebotomus caucasicus	74.14137271	39.81737831
Phlebotomus caucasicus	74.95990729	39.72219987
Phlebotomus caucasicus	75.32158537	41.37830473
Phlebotomus caucasicus	75.77844188	41.93033968
Phlebotomus caucasicus	76.73022628	42.57755308
Phlebotomus caucasicus	77.16804711	42.12069656
Phlebotomus caucasicus	77.09190436	41.54962592
Phlebotomus caucasicus	78.04368876	41.87323262
Phlebotomus caucasicus	78.57668802	42.40623188
Phlebotomus caucasicus	76.23529839	41.24505491
Phlebotomus caucasicus	78.1388672	44.95701408
Phlebotomus caucasicus	75.89265601	43.77680142
Phlebotomus caucasicus	78.72897353	44.11944381
Phlebotomus caucasicus	80.99422041	43.98619399
Phlebotomus caucasicus	82.55514683	44.95701408
Phlebotomus caucasicus	77.92114652	42.71318235
Phlebotomus caucasicus	84.78208438	46.36149094
Phlebotomus caucasicus	82.15682505	44.20094035
Phlebotomus celiae	-1.65	37.7
Phlebotomus celiae	-0.30693	38.21806
Phlebotomus celiae	0.84791	34.70206
Phlebotomus chabaudi	-4.379950341	32.61217916
Phlebotomus chabaudi	-5.712448504	32.89771448
Phlebotomus chabaudi	-6.164546095	32.11249234
Phlebotomus chabaudi	-4.713074881	31.66039475
Phlebotomus chabaudi	-5.141377862	30.99414567
Phlebotomus chabaudi	-6.450081416	31.27968099
Phlebotomus chabaudi	-7.401865818	31.16070794
Phlebotomus chabaudi	-7.592222698	30.16133432
Phlebotomus chabaudi	-8.567801711	29.25713914
Phlebotomus chabaudi	-7.7313	29.306133
Phlebotomus chabaudi	-6.545259856	30.28030737
Phlebotomus chabaudi	-5.783832334	30.44686964
Phlebotomus chabaudi	3.448476369	32.9453037
Phlebotomus chabaudi	-2.642943806	37.03797663
Phlebotomus chabaudi	6.425182087	34.91946318

species	lon	lat
Phlebotomus chabaudi	8.842714469	34.2532141
Phlebotomus chabaudi	10.74628327	32.82553749
Phlebotomus chabaudi	10.3846052	31.39786089
Phlebotomus chabaudi	10.34596275	35.4526528
Phlebotomus chabaudi	10.14608803	36.15221433
Phlebotomus chabaudi	9.717785048	36.66617791
Phlebotomus chabaudi	-1.1306544	37.9922399
Phlebotomus chabaudi	-1.1306544	37.9922399
Phlebotomus chabaudi	-2.302446	37.1035929
Phlebotomus chabaudi	-3.59855709	37.1773363
Phlebotomus chadlii	-7.596585485	30.71790417
Phlebotomus chadlii	-6.264087321	32.26455382
Phlebotomus chadlii	-5.383686749	31.05102871
Phlebotomus chadlii	-4.408107737	33.19254362
Phlebotomus chadlii	-3.194582624	32.33593765
Phlebotomus chadlii	-2.671101203	34.76298788
Phlebotomus chadlii	6.024639043	35.94082108
Phlebotomus chadlii	9.236911401	34.03725227
Phlebotomus chadlii	9.451062892	36.4643025
Phlebotomus chadlii	9.689008992	35.65528576
Phlebotomus chadlii	10.54561495	35.46492888
Phlebotomus chadlii	10.54323549	36.53330687
Phlebotomus duboscqi	0.46998	35.9961
Phlebotomus duboscqi	-15.67807716	19.69989039
Phlebotomus duboscqi	-14.28609247	19.66419847
Phlebotomus duboscqi	-14.78577928	17.55837548
Phlebotomus duboscqi	-16.67745078	14.56025461
Phlebotomus duboscqi	-15.60669333	14.0605678
Phlebotomus duboscqi	-11.10951203	14.5245627
Phlebotomus duboscqi	-9.503375852	15.20270908
Phlebotomus duboscqi	-10.39567373	13.70364865
Phlebotomus duboscqi	-6.291103494	17.05868867
Phlebotomus duboscqi	-3.114523051	16.91592101
Phlebotomus duboscqi	-0.401937505	16.27346654
Phlebotomus duboscqi	-1.508386872	15.73808781
Phlebotomus duboscqi	-2.864679646	15.05994142
Phlebotomus duboscqi	-3.899745183	14.31041121
Phlebotomus duboscqi	-7.326169032	13.70364865
Phlebotomus duboscqi	-8.147083079	12.13320439
Phlebotomus duboscqi	-7.112017541	11.16952268
Phlebotomus duboscqi	-5.720032853	11.13383076
Phlebotomus duboscqi	-5.720032853	12.52581545
Phlebotomus duboscqi	-5.006194551	13.56088099
Phlebotomus duboscqi	-4.57789157	11.41936609
Phlebotomus duboscqi	-3.685593693	12.49012354
Phlebotomus duboscqi	-2.721911985	13.38242141

species	lon	lat
Phlebotomus duboscqi	-1.437003042	13.66795674
Phlebotomus duboscqi	-2.293609004	12.31166396
Phlebotomus duboscqi	-1.151467722	12.59719928
Phlebotomus duboscqi	-3.007447306	11.455058
Phlebotomus duboscqi	-2.257917089	11.27659842
Phlebotomus duboscqi	-0.973008146	11.31229034
Phlebotomus duboscqi	0.169133137	11.70490141
Phlebotomus duboscqi	2.667567193	13.63226482
Phlebotomus duboscqi	4.844774013	15.55962824
Phlebotomus duboscqi	7.807202965	14.23902738
Phlebotomus duboscqi	9.377647229	12.09751247
Phlebotomus duboscqi	0.490360372	9.313543095
Phlebotomus duboscqi	14.49943704	11.46695531
Phlebotomus duboscqi	15.8200379	12.71617233
Phlebotomus duboscqi	0.758049736	6.755622514
Phlebotomus duboscqi	15.42742684	5.970400382
Phlebotomus duboscqi	18.51001857	9.678790359
Phlebotomus duboscqi	21.4248583	14.22950953
Phlebotomus duboscqi	33.06042262	4.491565366
Phlebotomus duboscqi	34.44051	4.824689907
Phlebotomus duboscqi	37.62898775	6.01442041
Phlebotomus duboscqi	38.58077215	7.584864674
Phlebotomus duboscqi	34.99968334	11.85599718
Phlebotomus duboscqi	34.60707227	12.92675463
Phlebotomus duboscqi	35.67782973	13.64059293
Phlebotomus duboscqi	36.39166803	14.35443124
Phlebotomus duboscqi	30.07419906	15.28242103
Phlebotomus duboscqi	34.10738546	15.88918358
Phlebotomus duboscqi	37.92642038	13.89043634
Phlebotomus duboscqi	61.27087707	27.74320174
Phlebotomus elgonensis	0.84791	34.70206
Phlebotomus guggisbergi	-1.40478	36.63648
Phlebotomus halepensis	32.67582444	37.27504361
Phlebotomus halepensis	35.73255	33.078567
Phlebotomus halepensis	36.55395	36.322233
Phlebotomus halepensis	36.55395	36.322233
Phlebotomus halepensis	43.44269711	42.35105854
Phlebotomus halepensis	43.8947947	41.68480946
Phlebotomus halepensis	44.79898989	42.017934
Phlebotomus halepensis	44.9074	41.082633
Phlebotomus halepensis	44.53724918	40.23333825
Phlebotomus halepensis	45.93135	41.182167
Phlebotomus halepensis	46.86912096	40.42369513
Phlebotomus halepensis	45.96945	39.278367
Phlebotomus halepensis	46.84532635	39.44811612
Phlebotomus halepensis	46.03630961	38.09182334

species	lon	lat
Phlebotomus halepensis	46.39322876	38.11561795
Phlebotomus halepensis	48.1162	39.012533
Phlebotomus halepensis	47.70193231	35.6171839
Phlebotomus halepensis	46.65496947	34.85575638
Phlebotomus halepensis	46.84532635	33.95156119
Phlebotomus halepensis	49.05822509	31.8457382
Phlebotomus halepensis	51.64588893	32.31568175
Phlebotomus halepensis	51.61614567	33.26746615
Phlebotomus halepensis	51.64588893	35.73615695
Phlebotomus halepensis	52.03255134	30.0551938
Phlebotomus halepensis	54.08483646	32.64285764
Phlebotomus halepensis	56.34532442	34.81411581
Phlebotomus halepensis	58.6652989	34.07053424
Phlebotomus halepensis	56.13712158	36.86640093
Phlebotomus halepensis	56.04789179	38.88894278
Phlebotomus halepensis	57.74325776	37.55049597
Phlebotomus halepensis	58.93298826	38.05613143
Phlebotomus halepensis	60.0243	37.062833
Phlebotomus halepensis	59.26016415	36.65819809
Phlebotomus halepensis	60.673	36.692333
Phlebotomus halepensis	48.15997856	31.84970397
Phlebotomus halepensis	48.3620209	37.63511
Phlebotomus halepensis	57.6789735	36.2148413
Phlebotomus halepensis	51.49707446	35.7330843
Phlebotomus halepensis	46.9348322	37.6337887
Phlebotomus halepensis	47.5495505	38.0058809
Phlebotomus halepensis	42.7831674	41.1112964
Phlebotomus halepensis	47.1338639	38.0841098
Phlebotomus halepensis	46.3210443	38.0679372
Phlebotomus halepensis	46.3210443	38.0679372
Phlebotomus halepensis	44.5701991	34.8566834
Phlebotomus halepensis	50.7306348	36.6030106
Phlebotomus halepensis	45.0665583	39.3471366
Phlebotomus kandelakii	35.94699571	34.27043945
Phlebotomus kandelakii	47.48262267	35.29836661
Phlebotomus kandelakii	46.91155203	34.32754652
Phlebotomus kandelakii	47.79671152	34.65591213
Phlebotomus kandelakii	48.42488923	34.45603741
Phlebotomus kandelakii	51.32307273	32.42397771
Phlebotomus kandelakii	51.77517033	32.92366452
Phlebotomus kandelakii	51.82275955	35.39830397
Phlebotomus kandelakii	53.29802537	35.4696878
Phlebotomus kandelakii	53.79771218	35.18415248
Phlebotomus kandelakii	54.33071145	36.23587424
Phlebotomus kandelakii	54.84467502	36.04551736
Phlebotomus kandelakii	49.59082512	36.33105268

species	lon	lat
Phlebotomus kandelakii	49.72407494	36.88308764
Phlebotomus kandelakii	47.83954182	37.81583635
Phlebotomus kandelakii	47.57304219	38.29172855
Phlebotomus kandelakii	46.67836485	37.96812185
Phlebotomus kandelakii	48.69614778	38.89135272
Phlebotomus kandelakii	49.93156394	40.14294921
Phlebotomus kandelakii	49.56036802	40.5284219
Phlebotomus kandelakii	49.07495798	40.99955518
Phlebotomus kandelakii	48.54671763	41.27081373
Phlebotomus kandelakii	49.01785091	40.00018155
Phlebotomus kandelakii	48.31828938	39.74319976
Phlebotomus kandelakii	48.48961057	40.27144011
Phlebotomus kandelakii	47.93281669	40.41420777
Phlebotomus kandelakii	47.39029958	40.81395722
Phlebotomus kandelakii	47.66155814	39.91452096
Phlebotomus kandelakii	47.3639	39.5129
Phlebotomus kandelakii	46.8095	39.173233
Phlebotomus kandelakii	46.0576	39.154567
Phlebotomus kandelakii	45.53925	39.550433
Phlebotomus kandelakii	46.36595	39.667033
Phlebotomus kandelakii	46.9619966	39.78603006
Phlebotomus kandelakii	47.11904103	40.34282394
Phlebotomus kandelakii	46.57652392	40.79968045
Phlebotomus kandelakii	46.34809566	40.28571687
Phlebotomus kandelakii	44.97055	39.847
Phlebotomus kandelakii	44.70626757	40.34282394
Phlebotomus kandelakii	45.03463319	40.74257339
Phlebotomus kandelakii	45.5562	40.942267
Phlebotomus kandelakii	46.40175	41.154167
Phlebotomus kandelakii	45.94834621	41.57062582
Phlebotomus kandelakii	45.94834621	41.57062582
Phlebotomus kandelakii	45.0574	41.3796
Phlebotomus kandelakii	44.39955	41.1775
Phlebotomus kandelakii	43.96387574	41.64200965
Phlebotomus kandelakii	44.66343727	41.94182173
Phlebotomus kandelakii	45.30589174	42.14169646
Phlebotomus kandelakii	44.50639284	42.44150854
Phlebotomus kandelakii	43.6537	42.6258
Phlebotomus kandelakii	46.09111387	43.16962361
Phlebotomus kandelakii	46.74784511	42.38440148
Phlebotomus kandelakii	47.51879048	42.14169646
Phlebotomus kandelakii	59.67664649	36.22397694
Phlebotomus kandelakii	60.93776082	37.22335056
Phlebotomus kandelakii	62.10369671	37.10437751
Phlebotomus kandelakii	62.84132963	37.05678829
Phlebotomus kandelakii	62.3178482	36.67607453

species	lon	lat
Phlebotomus kandelakii	62.29405359	36.05741467
Phlebotomus kandelakii	63.0078919	36.03362006
Phlebotomus kandelakii	63.43619488	35.60531708
Phlebotomus kandelakii	62.00851827	35.72429013
Phlebotomus kandelakii	62.11559402	35.14132218
Phlebotomus kandelakii	66.53075	38.085767
Phlebotomus kandelakii	67.9928627	38.13944305
Phlebotomus kandelakii	67.10056483	37.12817212
Phlebotomus kandelakii	67.13625674	36.59279339
Phlebotomus kandelakii	67.5169705	35.52203594
Phlebotomus kandelakii	68.17132228	37.30663169
Phlebotomus kandelakii	71.60161275	39.99914054
Phlebotomus kandelakii	72.38207596	39.99914054
Phlebotomus kandelakii	73.1910927	39.94203347
Phlebotomus langeroni	8.1483	34.18652
Phlebotomus langeroni	-3.7037902	40.4167754
Phlebotomus langeroni	4.2040933	36.6372932
Phlebotomus langeroni	-6.67003469	32.95825788
Phlebotomus langeroni	-0.25738728	35.05218357
Phlebotomus langeroni	12.05632343	32.26821419
Phlebotomus longicuspis	-7.6310791	31.5438441
Phlebotomus longicuspis	-7.6710889	31.5634338
Phlebotomus longicuspis	-7.4841222	31.5319623
Phlebotomus longicuspis	-7.6758373	31.2258103
Phlebotomus longicuspis	-7.9504327	31.3526424
Phlebotomus longicuspis	-8.015974	31.63589
Phlebotomus longicuspis	-8.8457977	31.1719751
Phlebotomus longicuspis	-9.365921	30.368317
Phlebotomus longicuspis	-7.5247222	31.0175
Phlebotomus longicuspis	-9.4799447	30.4854645
Phlebotomus longicuspis	-9.6584978	30.8661606
Phlebotomus longicuspis	-7.0434589	32.0772052
Phlebotomus longicuspis	-9.6035176	30.4751364
Phlebotomus longicuspis	-5.661571	32.9340471
Phlebotomus longicuspis	-8.0710715	31.6889538
Phlebotomus longicuspis	-8.0124308	31.6430891
Phlebotomus longicuspis	-6.5730004	31.9649107
Phlebotomus longicuspis	-8.0751645	30.6262829
Phlebotomus longicuspis	-9.5981072	30.4277547
Phlebotomus longicuspis	-9.705330575	28.49155083
Phlebotomus longicuspis	-8.099194397	28.24170742
Phlebotomus longicuspis	-8.670265038	30.38322233
Phlebotomus longicuspis	-8.092437085	29.07075044
Phlebotomus longicuspis	-7.331818222	29.05072416
Phlebotomus longicuspis	-6.61797992	29.13995395
Phlebotomus longicuspis	-7.093872122	30.03225183

species	lon	lat
Phlebotomus longicuspis	-6.171830982	30.12148161
Phlebotomus longicuspis	-5.636452255	29.31841353
Phlebotomus longicuspis	-8.253859362	31.60864474
Phlebotomus longicuspis	-7.42104801	31.43018517
Phlebotomus longicuspis	-6.677466446	31.28146885
Phlebotomus longicuspis	-5.993371406	31.10300928
Phlebotomus longicuspis	-5.33901963	30.89480644
Phlebotomus longicuspis	-4.892870691	31.31121212
Phlebotomus longicuspis	-3.962707839	30.584364
Phlebotomus longicuspis	-4.208775652	31.48967169
Phlebotomus longicuspis	-4.208775652	32.38196957
Phlebotomus longicuspis	-5.547222468	31.78710432
Phlebotomus longicuspis	-6.231317507	31.87633411
Phlebotomus longicuspis	-6.736952971	32.20350999
Phlebotomus longicuspis	-7.629250848	32.26299652
Phlebotomus longicuspis	-8.432318937	32.35222631
Phlebotomus longicuspis	-7.54002106	33.18503766
Phlebotomus longicuspis	-6.736952971	33.60144334
Phlebotomus longicuspis	-6.26106077	34.13682206
Phlebotomus longicuspis	-5.547222468	34.01784901
Phlebotomus longicuspis	-4.714411116	33.83938944
Phlebotomus longicuspis	-5.695938781	33.27426745
Phlebotomus longicuspis	-4.952357216	33.12555113
Phlebotomus longicuspis	-5.249789842	32.4414561
Phlebotomus longicuspis	-3.197504724	31.81684758
Phlebotomus longicuspis	-2.364693372	32.61991567
Phlebotomus longicuspis	-3.316477775	32.76863198
Phlebotomus longicuspis	-4.179032389	34.79117384
Phlebotomus longicuspis	-4.030316077	33.60144334
Phlebotomus longicuspis	-3.405707563	33.98810575
Phlebotomus longicuspis	-2.840585574	33.72041639
Phlebotomus longicuspis	-2.245720322	34.25579511
Phlebotomus longicuspis	-2.543152948	34.73168731
Phlebotomus longicuspis	-1.76325	35.010933
Phlebotomus longiductus	24.74925487	45.68983891
Phlebotomus longiductus	24.60648721	44.33354614
Phlebotomus longiductus	25.96277999	44.44062188
Phlebotomus longiductus	27.75308645	44.26025874
Phlebotomus longiductus	28.47644259	44.9645792
Phlebotomus longiductus	28.37255	45.4485
Phlebotomus longiductus	27.96247902	45.66889966
Phlebotomus longiductus	28.7359	45.898333
Phlebotomus longiductus	28.14095	46.361167
Phlebotomus longiductus	28.43837122	46.35418443
Phlebotomus longiductus	32.66429396	34.1332727
Phlebotomus longiductus	30.41808277	46.63971975

species	lon	lat
Phlebotomus longiductus	31.54118837	47.78186103
Phlebotomus longiductus	32.58815121	47.6676469
Phlebotomus longiductus	33.38765011	47.26789745
Phlebotomus longiductus	32.43586571	46.92525507
Phlebotomus longiductus	34.66304121	45.23107883
Phlebotomus longiductus	34.2061847	45.00265057
Phlebotomus longiductus	33.76836387	45.05975764
Phlebotomus longiductus	33.32578412	45.36432865
Phlebotomus longiductus	45.54035062	43.66579007
Phlebotomus longiductus	62.27272041	46.55921465
Phlebotomus longiductus	67.57891846	48.5817565
Phlebotomus longiductus	68.50690825	48.60555111
Phlebotomus longiductus	65.43740355	45.44086798
Phlebotomus longiductus	66.57954483	44.48908357
Phlebotomus longiductus	67.48374002	43.51350456
Phlebotomus longiductus	68.6258813	42.37136328
Phlebotomus longiductus	60.96401686	36.18476466
Phlebotomus longiductus	63.12932638	36.06579161
Phlebotomus longiductus	63.07815	35.484867
Phlebotomus longiductus	65.53258199	34.92365033
Phlebotomus longiductus	65.53258199	34.92365033
Phlebotomus longiductus	66.2702149	35.44713175
Phlebotomus longiductus	66.91266937	35.16159643
Phlebotomus longiductus	67.60271307	35.11400721
Phlebotomus longiductus	67.07923165	35.73266707
Phlebotomus longiductus	67.93583761	35.68507785
Phlebotomus longiductus	68.55449747	36.041997
Phlebotomus longiductus	67.53132924	36.35132693
Phlebotomus longiductus	66.98405321	36.99378141
Phlebotomus longiductus	68.03101605	37.32690595
Phlebotomus longiductus	66.12744724	38.58802028
Phlebotomus longiductus	67.1982047	38.89735021
Phlebotomus longiductus	68.18806047	39.29234074
Phlebotomus longiductus	68.88762201	39.02108218
Phlebotomus longiductus	68.98755937	38.07881562
Phlebotomus longiductus	69.55863001	37.73617324
Phlebotomus longiductus	70.22107196	38.10165845
Phlebotomus longiductus	69.5032	39.5746
Phlebotomus longiductus	69.25976971	36.7501246
Phlebotomus longiductus	69.84987604	36.43603575
Phlebotomus longiductus	70.36383962	37.05469561
Phlebotomus longiductus	70.6265	42.1166
Phlebotomus longiductus	70.5131	41.388167
Phlebotomus longiductus	70.6403	40.337833
Phlebotomus longiductus	70.95013881	39.49126368
Phlebotomus longiductus	71.10718324	38.83453244

species	lon	lat
Phlebotomus longiductus	71.60687005	38.40622946
Phlebotomus longiductus	71.56403975	37.77805175
Phlebotomus longiductus	71.56403975	37.77805175
Phlebotomus longiductus	72.93460929	40.00522725
Phlebotomus longiductus	71.54976298	40.77617262
Phlebotomus longiductus	72.39209218	40.87610998
Phlebotomus longiductus	73.44857287	40.66195849
Phlebotomus longiductus	73.3914658	41.23302913
Phlebotomus longiductus	73.89115261	41.68988565
Phlebotomus longiductus	72.62425	42.6531
Phlebotomus longiductus	73.43205	42.647
Phlebotomus longiductus	73.27725167	44.10265911
Phlebotomus longiductus	73.80549202	43.3888208
Phlebotomus longiductus	74.49325	43.160167
Phlebotomus longiductus	75.23316862	43.11756225
Phlebotomus longiductus	76.3753099	43.70290966
Phlebotomus longiductus	76.60373816	45.0306489
Phlebotomus longiductus	78.55687907	45.7873175
Phlebotomus longiductus	78.97328474	44.76414927
Phlebotomus longiductus	80.43665326	46.5011558
Phlebotomus longiductus	81.97140561	47.60760517
Phlebotomus longiductus	83.31580108	48.38092999
Phlebotomus longipes	38.02703043	12.9447874
Phlebotomus longipes	39.15727441	11.72531363
Phlebotomus longipes	39.81162619	11.54685405
Phlebotomus longipes	39.90085597	10.80327249
Phlebotomus longipes	39.18701767	10.53558313
Phlebotomus longipes	35.826029	9.464825674
Phlebotomus longipes	36.74807014	7.828946233
Phlebotomus longipes	37.49165171	6.698702255
Phlebotomus longipes	38.65163895	8.988933473
Phlebotomus longipes	37.84857086	8.72124411
Phlebotomus longipes	37.96754391	7.828946233
Phlebotomus longipes	38.44343611	7.293567507
Phlebotomus longipes	38.74086873	8.126378859
Phlebotomus longipes	39.42496377	8.542784535
Phlebotomus martini	37.39286529	-1.788318257
Phlebotomus martini	40.21014712	-0.512927158
Phlebotomus martini	39.2773984	-1.46471156
Phlebotomus martini	38.40175675	-1.502782936
Phlebotomus martini	38.78247051	-3.082745044
Phlebotomus martini	40.14495	-2.797
Phlebotomus martini	36.55325109	0.929120086
Phlebotomus martini	35.73233705	0.143897954
Phlebotomus martini	42.37103325	9.495179707
Phlebotomus martini	39.33722047	12.81452781

species	lon	lat
Phlebotomus martini	38.19507919	12.314841
Phlebotomus martini	37.30278131	11.49392695
Phlebotomus martini	35.16126641	12.63606824
Phlebotomus martini	34.83935	10.7161
Phlebotomus martini	33.76928172	11.56531078
Phlebotomus martini	33.44805448	4.355543935
Phlebotomus martini	34.3427	4.525733
Phlebotomus martini	32.5557566	2.535256265
Phlebotomus martini	33.41236257	0.929120086
Phlebotomus martini	35.08988258	3.677397548
Phlebotomus martini	36.0755	4.439367
Phlebotomus martini	35.94648854	3.213402652
Phlebotomus martini	36.51755918	2.49956435
Phlebotomus martini	38.65907408	3.034943077
Phlebotomus martini	39.62275579	3.106326907
Phlebotomus martini	41.0124	1.218533
Phlebotomus martini	40.08675069	0.714968596
Phlebotomus martini	39.26583664	-0.034561621
Phlebotomus martini	38.37353876	0.108206039
Phlebotomus martini	36.41048343	-0.605632262
Phlebotomus martini	36.51755918	0.036822209
Phlebotomus martini	37.16001365	0.036822209
Phlebotomus martini	37.65970046	-0.712708008
Phlebotomus mascittii	46.58472	0.34389
Phlebotomus mascittii	46.71583	0.15639
Phlebotomus mascittii	2.274108887	49.79370117
Phlebotomus mascittii	2.720275879	49.16912842
Phlebotomus mascittii	3.047485352	48.51470947
Phlebotomus mascittii	0.876098633	46.55169678
Phlebotomus mascittii	-0.402770996	45.89727783
Phlebotomus mascittii	0.667907715	45.5402832
Phlebotomus mascittii	1.471130371	45.92712402
Phlebotomus mascittii	0.132507324	43.45831299
Phlebotomus mascittii	0.965270996	43.27990723
Phlebotomus mascittii	2.155090332	43.87469482
Phlebotomus mascittii	2.987915039	43.87469482
Phlebotomus mascittii	3.141723633	43.39532471
Phlebotomus mascittii	3.75567627	43.88067627
Phlebotomus mascittii	4.455078125	44.52307129
Phlebotomus mascittii	4.483703613	43.90930176
Phlebotomus mascittii	4.526489258	43.5333252
Phlebotomus mascittii	6.182678223	43.28112793
Phlebotomus mascittii	6.93927002	43.65228271
Phlebotomus mascittii	4.497924805	45.65112305
Phlebotomus mascittii	5.368896484	45.69390869
Phlebotomus mascittii	6.639526367	46.62188721

species	lon	lat
Phlebotomus mascittii	8.652526855	46.37908936
Phlebotomus mascittii	7.116088867	48.74908447
Phlebotomus mascittii	5.123291016	48.48132324
Phlebotomus mascittii	5.063720703	47.2321167
Phlebotomus mascittii	1.613525391	45.03112793
Phlebotomus mascittii	12.35089111	42.65167236
Phlebotomus mascittii	12.98168945	41.69909668
Phlebotomus mascittii	13.79547119	41.55627441
Phlebotomus mascittii	13.9666748	42.48431396
Phlebotomus mascittii	14.60931396	42.01312256
Phlebotomus mascittii	14.26647949	41.19927979
Phlebotomus mascittii	14.92327881	41.05651855
Phlebotomus mascittii	15.23748779	40.41412354
Phlebotomus mascittii	15.85131836	40.72808838
Phlebotomus mascittii	20.91149902	42.13409424
Phlebotomus mascittii	8.928710938	42.27172852
Phlebotomus mascittii	8.776489258	39.96832275
Phlebotomus mascittii	8.795288086	39.20690918
Phlebotomus mascittii	7.6233329	47.9170636
Phlebotomus mascittii	7.5591687	47.8140501
Phlebotomus mascittii	7.5301043	47.6613376
Phlebotomus mascittii	7.6678713	47.9026752
Phlebotomus mascittii	7.673928	47.9243593
Phlebotomus mascittii	7.6482455	48.0429731
Phlebotomus mascittii	7.7118623	48.0254467
Phlebotomus mascittii	7.638857	47.735372
Phlebotomus mascittii	9.4713346	48.1596486
Phlebotomus mascittii	8.0918589	48.6391462
Phlebotomus mascittii	7.7416927	48.2924014
Phlebotomus mascittii	8.2285242	48.76564
Phlebotomus mascittii	7.5771823	47.7558933
Phlebotomus mascittii	5.3923682	43.2846279
Phlebotomus mascittii	5.36978	43.296482
Phlebotomus mascittii	3.5787477	43.6975255
Phlebotomus mascittii	3.6673942	43.9755057
Phlebotomus mascittii	0.68484	47.394144
Phlebotomus mascittii	4.2040933	36.6372932
Phlebotomus mongolensis	56.3669378	48.84539046
Phlebotomus mongolensis	55.79586716	48.39329287
Phlebotomus mongolensis	55.03443963	47.89360605
Phlebotomus mongolensis	54.4871636	47.5366869
Phlebotomus mongolensis	53.70194147	47.15597314
Phlebotomus mongolensis	53.01189778	47.63186534
Phlebotomus mongolensis	52.1077026	47.56048151
Phlebotomus mongolensis	52.20288104	44.65753909
Phlebotomus mongolensis	53.27363849	43.42021936

species	lon	lat
Phlebotomus mongolensis	54.34439594	39.77964402
Phlebotomus mongolensis	55.48653722	38.94683267
Phlebotomus mongolensis	56.39073241	39.11339494
Phlebotomus mongolensis	56.96180305	39.61308175
Phlebotomus mongolensis	57.69943596	39.27995721
Phlebotomus mongolensis	56.81015	38.333467
Phlebotomus mongolensis	55.859	38.1731
Phlebotomus mongolensis	50.9417667	35.63938187
Phlebotomus mongolensis	50.32310684	33.75960768
Phlebotomus mongolensis	51.67939962	33.94996456
Phlebotomus mongolensis	51.60801579	32.80782328
Phlebotomus mongolensis	57.96117667	34.85415974
Phlebotomus mongolensis	57.86599823	37.1146477
Phlebotomus mongolensis	57.84220362	38.35196742
Phlebotomus mongolensis	59.07952334	38.25678898
Phlebotomus mongolensis	59.81715626	36.66255011
Phlebotomus mongolensis	61.31621669	36.8767016
Phlebotomus mongolensis	62.14902804	37.42397763
Phlebotomus mongolensis	63.10081244	37.37638841
Phlebotomus mongolensis	62.93425017	36.56737167
Phlebotomus mongolensis	62.03005499	36.25804174
Phlebotomus mongolensis	63.79085614	36.30563096
Phlebotomus mongolensis	60.57858378	42.94432716
Phlebotomus mongolensis	59.07952334	42.99191638
Phlebotomus mongolensis	57.94699642	45.49502853
Phlebotomus mongolensis	62.79148251	46.69693546
Phlebotomus mongolensis	64.94489472	46.28052978
Phlebotomus mongolensis	64.8973055	45.54289687
Phlebotomus mongolensis	65.95616565	44.93613431
Phlebotomus mongolensis	66.663163	45.62141908
Phlebotomus mongolensis	67.80530428	43.86061794
Phlebotomus mongolensis	72.37386942	46.01403015
Phlebotomus mongolensis	73.43272956	44.12235865
Phlebotomus mongolensis	70.26804643	41.81428147
Phlebotomus mongolensis	69.5066189	40.91008629
Phlebotomus mongolensis	72.48005	41.0901
Phlebotomus mongolensis	73.58739453	41.26700544
Phlebotomus mongolensis	72.0804	39.361033
Phlebotomus mongolensis	71.81707554	38.82091953
Phlebotomus mongolensis	70.23235451	39.16356191
Phlebotomus mongolensis	69.09021323	39.44909723
Phlebotomus mongolensis	68.7613	40.169133
Phlebotomus mongolensis	67.34844777	39.76318609
Phlebotomus mongolensis	66.86303773	39.13500838
Phlebotomus mongolensis	67.61970633	39.02079425
Phlebotomus mongolensis	66.69171653	38.52110744

species	lon	lat
Phlebotomus mongolensis	66.558	37.880333
Phlebotomus mongolensis	67.43410837	37.9072065
Phlebotomus mongolensis	67.24851041	36.93638641
Phlebotomus mongolensis	70.44769573	34.42486532
Phlebotomus mongolensis	71.05683775	35.35761403
Phlebotomus mongolensis	71.4877	35.833933
Phlebotomus mongolensis	71.71015	35.232033
Phlebotomus mongolensis	52.3752135	30.38929945
Phlebotomus mongolensis	53.46024772	30.29412101
Phlebotomus mongolensis	53.99324698	29.01872991
Phlebotomus mongolensis	55.04020983	29.15197973
Phlebotomus mongolensis	76.14920156	44.73848042
Phlebotomus mongolensis	77.51739164	45.79734057
Phlebotomus mongolensis	82.72841124	46.24943816
Phlebotomus mongolensis	80.83673974	44.63140467
Phlebotomus mongolensis	98.77728086	43.21235362
Phlebotomus mongolensis	100.3762787	43.34084451
Phlebotomus mongolensis	99.5196727	43.91191515
Phlebotomus mongolensis	99.61961006	44.78279788
Phlebotomus mongolensis	99.61961006	45.63940384
Phlebotomus mongolensis	102.1180441	43.85480809
Phlebotomus mongolensis	103.6599348	45.22537763
Phlebotomus mongolensis	107.0435284	45.31103822
Phlebotomus mongolensis	107.891	42.4127
Phlebotomus mongolensis	106.6152254	42.0416588
Phlebotomus mongolensis	117.3775275	39.39569816
Phlebotomus mongolensis	116.6208589	40.03815263
Phlebotomus mongolensis	115.6357621	38.96739518
Phlebotomus mongolensis	112.1950615	38.6247528
Phlebotomus mongolensis	111.4098393	36.08348844
Phlebotomus mongolensis	111.7524817	34.72719567
Phlebotomus mongolensis	114.3080228	34.54159771
Phlebotomus mongolensis	115.5643783	36.39757729
Phlebotomus mongolensis	116.5923054	35.6409087
Phlebotomus mongolensis	116.192556	35.05556129
Phlebotomus mongolensis	117.0348852	34.61298154
Phlebotomus mongolensis	118.6196062	34.37027652
Phlebotomus mongolensis	118.7480971	35.12694512
Phlebotomus mongolensis	112.1950615	33.05681404
Phlebotomus mongolensis	106.2502161	36.41185406
Phlebotomus mongolensis	109.2340106	42.66289641
Phlebotomus neglectus	19.089904	42.0912106
Phlebotomus neglectus	19.7431234	41.3715563
Phlebotomus neglectus	19.6877385	41.8217526
Phlebotomus neglectus	17.1204087	40.8544104
Phlebotomus neglectus	8.950409418	40.15059207

species	lon	lat
Phlebotomus neglectus	14.56593739	41.00719803
Phlebotomus neglectus	15.66048945	41.36411718
Phlebotomus neglectus	14.94665115	41.863804
Phlebotomus neglectus	13.15015809	37.96148795
Phlebotomus neglectus	13.84020178	37.48559575
Phlebotomus neglectus	14.92285654	37.97338525
Phlebotomus neglectus	14.85147271	37.16436851
Phlebotomus neglectus	16.36243045	39.99592711
Phlebotomus neglectus	16.54089003	39.35347263
Phlebotomus neglectus	16.31484123	38.53255859
Phlebotomus neglectus	17.56405826	40.60268966
Phlebotomus neglectus	28.17253815	44.30646943
Phlebotomus neglectus	28.66746604	45.01078989
Phlebotomus neglectus	23.88950834	44.11611255
Phlebotomus neglectus	18.86408669	44.53489769
Phlebotomus neglectus	21.12933357	44.87754008
Phlebotomus neglectus	22.01095	44.6081
Phlebotomus neglectus	22.5707	44.4057
Phlebotomus neglectus	22.34235	43.9198
Phlebotomus neglectus	21.52908302	44.02093411
Phlebotomus neglectus	20.74861981	43.96382705
Phlebotomus neglectus	19.70165697	43.43082778
Phlebotomus neglectus	20.52019155	43.48793485
Phlebotomus neglectus	21.16740495	43.39275641
Phlebotomus neglectus	21.98593953	43.43082778
Phlebotomus neglectus	22.42695	42.8726
Phlebotomus neglectus	21.58619008	42.87879283
Phlebotomus neglectus	20.88186963	42.84072146
Phlebotomus neglectus	19.98719229	42.82168577
Phlebotomus neglectus	19.3399789	42.99300696
Phlebotomus neglectus	18.9918	43.277267
Phlebotomus neglectus	18.12169486	43.50697054
Phlebotomus neglectus	17.43641009	43.84961292
Phlebotomus neglectus	21.523849	42.377276
Phlebotomus neglectus	21.3958332	41.79375861
Phlebotomus neglectus	22.11918935	41.71761586
Phlebotomus neglectus	21.12395	40.954967
Phlebotomus neglectus	21.31969045	40.25186788
Phlebotomus neglectus	21.2435477	39.28104779
Phlebotomus neglectus	22.5819	40.4299
Phlebotomus neglectus	23.50879458	40.5374032
Phlebotomus neglectus	25.39	41.2841
Phlebotomus neglectus	19.77589615	41.14654522
Phlebotomus neglectus	19.57225	41.8667
Phlebotomus neglectus	19.3078	42.254267
Phlebotomus neglectus	18.55455	42.756833

species	lon	lat
Phlebotomus neglectus	18.16738051	42.96445343
Phlebotomus neglectus	17.61534556	43.31661366
Phlebotomus neglectus	16.96030489	43.70870209
Phlebotomus neglectus	16.22574033	43.89720214
Phlebotomus neglectus	15.68988571	44.28743375
Phlebotomus neglectus	15.35676117	44.85850439
Phlebotomus neglectus	14.86183328	45.38198581
Phlebotomus neglectus	13.92908457	45.27728953
Phlebotomus neglectus	22.41424251	37.48693419
Phlebotomus neglectus	22.9259	37.1296
Phlebotomus neglectus	24.03925575	38.56562318
Phlebotomus neglectus	27.84639336	40.08847823
Phlebotomus neglectus	30.62560381	37.72805291
Phlebotomus neglectus	30.51138969	37.08083951
Phlebotomus neglectus	31.46317409	37.32830346
Phlebotomus neglectus	32.09135179	37.06180383
Phlebotomus neglectus	34.19289175	37.15698227
Phlebotomus neglectus	36.36296019	36.9475897
Phlebotomus neglectus	37.01017359	36.41459043
Phlebotomus neglectus	36.3439245	35.80544841
Phlebotomus neglectus	36.43910294	35.04402089
Phlebotomus neglectus	35.63960405	34.56812869
Phlebotomus neglectus	36.21067469	33.9209153
Phlebotomus neglectus	36.53428138	33.48309447
Phlebotomus neglectus	35.35406873	33.5973086
Phlebotomus neglectus	35.8680323	33.08334502
Phlebotomus neglectus	35.12564047	32.6455242
Phlebotomus neglectus	35.14467616	31.99831081
Phlebotomus neglectus	33.44970677	45.16386855
Phlebotomus neglectus	34.23016998	45.33518974
Phlebotomus neglectus	34.05884878	44.63086929
Phlebotomus neglectus	34.85834768	44.93544029
Phlebotomus neglectus	35.63881089	45.10676149
Phlebotomus neglectus	43.07581627	37.14794031
Phlebotomus neglectus	44.64626053	36.57686967
Phlebotomus neglectus	50.64607146	36.07718286
Phlebotomus neglectus	24.8811388	35.29662051
Phlebotomus neglectus	25.73060638	35.19192422
Phlebotomus neglectus	23.79134566	35.41797302
Phlebotomus orientalis	5.43011939	14.39370312
Phlebotomus orientalis	17.38691094	8.897148193
Phlebotomus orientalis	24.41821822	13.71555673
Phlebotomus orientalis	44.26054354	17.8383695
Phlebotomus orientalis	43.6894729	15.36373005
Phlebotomus orientalis	43.85603517	13.65051813
Phlebotomus orientalis	41.85728793	9.629229029

species	lon	lat
Phlebotomus orientalis	38.93055089	7.630481784
Phlebotomus orientalis	34.99968131	6.797670432
Phlebotomus orientalis	29.28897489	11.98489542
Phlebotomus orientalis	29.38415334	10.36686194
Phlebotomus orientalis	31.21633831	9.248515268
Phlebotomus orientalis	32.19191732	9.84338052
Phlebotomus orientalis	31.81120356	11.29485173
Phlebotomus orientalis	33.66718315	10.22409428
Phlebotomus orientalis	34.52378911	11.62797627
Phlebotomus orientalis	33.7861562	12.72252834
Phlebotomus orientalis	33.00093406	13.48395586
Phlebotomus orientalis	34.54758372	14.22158877
Phlebotomus orientalis	34.71414599	15.41131927
Phlebotomus orientalis	35.689725	16.10136297
Phlebotomus orientalis	36.584754	14.468884
Phlebotomus orientalis	37.33155309	12.6273499
Phlebotomus orientalis	38.09298062	12.10386848
Phlebotomus orientalis	33.10800981	3.097608568
Phlebotomus orientalis	36.64745806	0.123282311
Phlebotomus orientalis	38.31308076	-1.601826918
Phlebotomus orientalis	39.62178431	1.164296501
Phlebotomus orientalis	40.48433893	2.056594378
Phlebotomus orientalis	35.1497	5.698367
Phlebotomus papatasi	-8.77792226	38.75729207
Phlebotomus papatasi	-6.703032263	39.1951129
Phlebotomus papatasi	-6.588818135	40.28014712
Phlebotomus papatasi	-5.656069421	40.08979024
Phlebotomus papatasi	-6.322318502	37.29154409
Phlebotomus papatasi	-5.084998779	37.38672253
Phlebotomus papatasi	-5.427641164	41.2700029
Phlebotomus papatasi	-4.742356395	40.24207574
Phlebotomus papatasi	-3.752500616	40.6227895
Phlebotomus papatasi	-4.152250065	39.48064822
Phlebotomus papatasi	-4.133214377	38.64307795
Phlebotomus papatasi	-2.895894654	38.62404226
Phlebotomus papatasi	-3.505036671	37.53900804
Phlebotomus papatasi	-2.267716948	37.29154409
Phlebotomus papatasi	-1.525325115	37.90068611
Phlebotomus papatasi	-0.630647776	38.62404226
Phlebotomus papatasi	-4.337848024	36.89179465
Phlebotomus papatasi	-5.494266072	36.4063846
Phlebotomus papatasi	-8.863582857	32.22329215
Phlebotomus papatasi	-7.531084693	33.19887116
Phlebotomus papatasi	-6.841041002	33.67476337
Phlebotomus papatasi	-6.05581887	33.9840933
Phlebotomus papatasi	-9.339475058	30.03418803

species	lon	lat
Phlebotomus papatasi	-8.673225976	30.22454491
Phlebotomus papatasi	-9.720188819	29.17758206
Phlebotomus papatasi	-9.101528957	29.24896589
Phlebotomus papatasi	-8.363896045	29.34414433
Phlebotomus papatasi	-9.006350517	28.67789525
Phlebotomus papatasi	-7.626263134	29.7248581
Phlebotomus papatasi	-8.340101435	31.3904808
Phlebotomus papatasi	-7.531084693	31.77119456
Phlebotomus papatasi	-6.793451782	31.34289158
Phlebotomus papatasi	-6.674478731	32.03293527
Phlebotomus papatasi	-6.19858653	31.72360534
Phlebotomus papatasi	-5.556132058	32.03293527
Phlebotomus papatasi	-5.341980568	30.31972335
Phlebotomus papatasi	-4.461579996	30.79561555
Phlebotomus papatasi	-5.127829077	32.43744364
Phlebotomus papatasi	-4.461579996	31.24771314
Phlebotomus papatasi	-3.771536304	31.27150775
Phlebotomus papatasi	-3.533590204	31.91396222
Phlebotomus papatasi	-3.890509354	32.98471967
Phlebotomus papatasi	-4.580553046	33.48440649
Phlebotomus papatasi	-4.461579996	34.26962862
Phlebotomus papatasi	-2.819751902	33.9840933
Phlebotomus papatasi	-2.462832751	34.67413699
Phlebotomus papatasi	-1.86925	34.714467
Phlebotomus papatasi	-2.12970821	34.26962862
Phlebotomus papatasi	-1.439664518	34.24583401
Phlebotomus papatasi	-2.343859701	33.55579032
Phlebotomus papatasi	-2.898	31.5417
Phlebotomus papatasi	-3.224260273	30.51008023
Phlebotomus papatasi	-3.295644103	29.55829583
Phlebotomus papatasi	-2.748368072	30.10557186
Phlebotomus papatasi	-2.22488665	29.67726888
Phlebotomus papatasi	-2.0678	31.8524
Phlebotomus papatasi	-0.963772317	32.08052449
Phlebotomus papatasi	-1.320691468	32.88954123
Phlebotomus papatasi	2.200910821	34.31721784
Phlebotomus papatasi	4.366220336	34.31721784
Phlebotomus papatasi	5.436977788	34.38860167
Phlebotomus papatasi	6.22219992	34.48378011
Phlebotomus papatasi	6.24599453	35.8876621
Phlebotomus papatasi	7.102600493	36.38734891
Phlebotomus papatasi	7.554698084	35.74489444
Phlebotomus papatasi	7.697465744	35.10243997
Phlebotomus papatasi	7.846419923	34.51778862
Phlebotomus papatasi	7.031216662	33.43681727
Phlebotomus papatasi	9.077553127	35.74489444

species	lon	lat
Phlebotomus papatasi	9.720007599	36.93462494
Phlebotomus papatasi	10.07692675	36.3635543
Phlebotomus papatasi	10.46075	36.089633
Phlebotomus papatasi	10.73936869	36.61530128
Phlebotomus papatasi	10.1468829	36.8580063
Phlebotomus papatasi	9.568277302	33.73208855
Phlebotomus papatasi	10.5225	33.566967
Phlebotomus papatasi	10.79607918	32.91831288
Phlebotomus papatasi	10.53909739	32.33296548
Phlebotomus papatasi	12.56639817	32.30441194
Phlebotomus papatasi	9.278458952	40.57779786
Phlebotomus papatasi	9.335566016	39.60697777
Phlebotomus papatasi	9.021477163	42.33003295
Phlebotomus papatasi	-0.591545301	41.79703368
Phlebotomus papatasi	0.207953597	41.64474818
Phlebotomus papatasi	0.265060662	40.90235634
Phlebotomus papatasi	0.969381119	41.4543913
Phlebotomus papatasi	1.978272586	41.4543913
Phlebotomus papatasi	3.679587205	43.66729003
Phlebotomus papatasi	4.229242697	44.00279403
Phlebotomus papatasi	4.814590105	43.76722739
Phlebotomus papatasi	5.285723384	43.50310722
Phlebotomus papatasi	6.035253601	43.47455369
Phlebotomus papatasi	8.544395231	45.20918076
Phlebotomus papatasi	9.8887907	45.38764034
Phlebotomus papatasi	10.04345567	44.76898048
Phlebotomus papatasi	10.92385624	45.17348885
Phlebotomus papatasi	11.95892177	45.22107807
Phlebotomus papatasi	11.56631071	44.36447211
Phlebotomus papatasi	10.97144546	43.37699579
Phlebotomus papatasi	12.19686788	43.78150416
Phlebotomus papatasi	11.3164673	42.78213054
Phlebotomus papatasi	11.94702447	42.86541167
Phlebotomus papatasi	12.76793852	42.03260032
Phlebotomus papatasi	13.07726845	42.50849252
Phlebotomus papatasi	13.38659838	42.04449762
Phlebotomus papatasi	14.04095015	42.16347067
Phlebotomus papatasi	13.76731214	41.65188656
Phlebotomus papatasi	14.50494505	41.68757847
Phlebotomus papatasi	14.83806959	40.99753478
Phlebotomus papatasi	15.42103754	44.55482899
Phlebotomus papatasi	15.06411839	45.42333225
Phlebotomus papatasi	13.50795089	37.7257759
Phlebotomus papatasi	15.09267192	37.82571326
Phlebotomus papatasi	14.87852043	37.04049113
Phlebotomus papatasi	16.34902733	38.69659599

species	lon	lat
Phlebotomus papatasi	16.46324146	39.49609489
Phlebotomus papatasi	17.93374836	40.49546851
Phlebotomus papatasi	17.41343955	43.66966949
Phlebotomus papatasi	17.9964075	43.31275034
Phlebotomus papatasi	18.25814821	43.90761559
Phlebotomus papatasi	18.72735	42.966767
Phlebotomus papatasi	19.1742407	42.86065275
Phlebotomus papatasi	18.91249999	44.45489162
Phlebotomus papatasi	18.56747814	45.35908681
Phlebotomus papatasi	18.9847	45.768833
Phlebotomus papatasi	19.55495446	45.51375177
Phlebotomus papatasi	19.67392751	46.07292511
Phlebotomus papatasi	20.14981971	43.52690183
Phlebotomus papatasi	19.1742407	43.57449105
Phlebotomus papatasi	20.63760922	43.96710212
Phlebotomus papatasi	21.81544241	43.83623176
Phlebotomus papatasi	21.16109064	43.455518
Phlebotomus papatasi	21.3157556	42.52752821
Phlebotomus papatasi	21.99390199	42.9915231
Phlebotomus papatasi	22.6251	43.4977
Phlebotomus papatasi	23.74280583	44.26453474
Phlebotomus papatasi	22.57005	40.599867
Phlebotomus papatasi	19.70961942	41.13554352
Phlebotomus papatasi	20.97073376	39.25576933
Phlebotomus papatasi	25.13479052	41.83748452
Phlebotomus papatasi	25.47981236	41.13554352
Phlebotomus papatasi	26.2650345	42.15871175
Phlebotomus papatasi	26.20554797	43.89571829
Phlebotomus papatasi	22.50548611	37.5425574
Phlebotomus papatasi	21.97248684	37.27605777
Phlebotomus papatasi	21.13372684	32.38269621
Phlebotomus papatasi	25.05190596	35.3205374
Phlebotomus papatasi	27.69191795	39.90575876
Phlebotomus papatasi	28.83405923	39.96286582
Phlebotomus papatasi	27.05498382	38.27906404
Phlebotomus papatasi	28.47552204	37.33679748
Phlebotomus papatasi	26.14721945	29.7629731
Phlebotomus papatasi	27.6938691	30.97649821
Phlebotomus papatasi	30.83475763	39.06666563
Phlebotomus papatasi	32.73832644	37.82934591
Phlebotomus papatasi	27.12279846	45.46741573
Phlebotomus papatasi	28.36071305	46.73388385
Phlebotomus papatasi	28.68907867	46.32699602
Phlebotomus papatasi	28.32502114	45.98435364
Phlebotomus papatasi	28.97461399	45.86300113
Phlebotomus papatasi	28.56058777	45.48466683

species	lon	lat
Phlebotomus papatasi	28.97461399	45.34189917
Phlebotomus papatasi	33.19458808	40.17846878
Phlebotomus papatasi	31.4841522	24.64614049
Phlebotomus papatasi	33.60187249	25.19341652
Phlebotomus papatasi	34.00638086	24.38439978
Phlebotomus papatasi	33.1497749	19.62547777
Phlebotomus papatasi	34.19673774	20.10136997
Phlebotomus papatasi	37.02353742	18.98778222
Phlebotomus papatasi	31.54125926	26.92566413
Phlebotomus papatasi	32.32172247	29.17187532
Phlebotomus papatasi	32.03618715	30.18076679
Phlebotomus papatasi	32.49304366	30.52340918
Phlebotomus papatasi	31.44608082	31.13255119
Phlebotomus papatasi	30.66561761	30.88508725
Phlebotomus papatasi	32.22892349	30.88556314
Phlebotomus papatasi	33.67087686	30.93553182
Phlebotomus papatasi	33.11146558	16.38488982
Phlebotomus papatasi	32.96274927	14.74901038
Phlebotomus papatasi	34.24170956	14.98695648
Phlebotomus papatasi	35.55041311	14.98695648
Phlebotomus papatasi	30.43457195	12.22083306
Phlebotomus papatasi	31.53507266	9.543939433
Phlebotomus papatasi	32.81403295	10.94187277
Phlebotomus papatasi	32.99249253	12.31006285
Phlebotomus papatasi	37.27552234	15.70079479
Phlebotomus papatasi	38.28679327	15.70079479
Phlebotomus papatasi	35.7526673	12.78892938
Phlebotomus papatasi	39.28497716	45.20333522
Phlebotomus papatasi	32.48923653	46.97365421
Phlebotomus papatasi	32.60345065	46.5167977
Phlebotomus papatasi	33.70752056	45.35562072
Phlebotomus papatasi	34.19848268	45.78273397
Phlebotomus papatasi	33.90342952	44.81191388
Phlebotomus papatasi	34.50305369	45.06889567
Phlebotomus papatasi	35.10267786	45.25925255
Phlebotomus papatasi	30.30568448	46.62030425
Phlebotomus papatasi	33.10234431	45.44960943
Phlebotomus papatasi	37.07445788	45.20055918
Phlebotomus papatasi	37.66694367	45.10062182
Phlebotomus papatasi	38.34509006	44.80794812
Phlebotomus papatasi	35.87520954	45.39329552
Phlebotomus papatasi	38.93043747	40.78546929
Phlebotomus papatasi	40.31052485	40.73788007
Phlebotomus papatasi	41.98804486	41.70156177
Phlebotomus papatasi	41.8333799	42.42729738
Phlebotomus papatasi	44.2009436	42.09417284

species	lon	lat
Phlebotomus papatasi	44.10576516	40.85685312
Phlebotomus papatasi	44.450787	40.42855013
Phlebotomus papatasi	45.05754956	40.45234474
Phlebotomus papatasi	45.22411183	41.80863752
Phlebotomus papatasi	46.02123127	41.34464262
Phlebotomus papatasi	32.96433557	34.86299084
Phlebotomus papatasi	33.73528094	35.21990999
Phlebotomus papatasi	34.69896265	37.01878251
Phlebotomus papatasi	36.22657661	36.74038558
Phlebotomus papatasi	37.49720879	36.11220787
Phlebotomus papatasi	36.1908847	35.23418676
Phlebotomus papatasi	37.21167347	35.18421808
Phlebotomus papatasi	36.29796044	34.7130848
Phlebotomus papatasi	36.4407281	33.97783135
Phlebotomus papatasi	35.93033372	34.28835101
Phlebotomus papatasi	35.50916912	33.10337943
Phlebotomus papatasi	36.11593168	33.38177637
Phlebotomus papatasi	35.65907516	32.610831
Phlebotomus papatasi	36.13020844	32.88922794
Phlebotomus papatasi	36.49245	32.435767
Phlebotomus papatasi	37.20039425	32.57059349
Phlebotomus papatasi	35.45745922	31.55242575
Phlebotomus papatasi	36.26774129	31.23740611
Phlebotomus papatasi	39.68940621	25.6361549
Phlebotomus papatasi	45.45721969	26.41661811
Phlebotomus papatasi	46.69453942	25.50290508
Phlebotomus papatasi	49.39760712	25.40772664
Phlebotomus papatasi	46.84682492	24.3607638
Phlebotomus papatasi	43.23004419	24.3607638
Phlebotomus papatasi	40.24144117	21.77191023
Phlebotomus papatasi	41.44068951	20.30616225
Phlebotomus papatasi	43.42040107	20.43941206
Phlebotomus papatasi	43.04206677	15.15938809
Phlebotomus papatasi	45.75465232	14.11718417
Phlebotomus papatasi	48.33874697	14.78819217
Phlebotomus papatasi	49.02403174	15.50203048
Phlebotomus papatasi	49.1667994	16.44429703
Phlebotomus papatasi	57.474	23.833333
Phlebotomus papatasi	47.73848827	29.75888835
Phlebotomus papatasi	47.87966963	30.7423989
Phlebotomus papatasi	44.96720936	32.47464652
Phlebotomus papatasi	44.96720936	32.89343165
Phlebotomus papatasi	44.9767272	33.46450229
Phlebotomus papatasi	44.4151744	33.25510973
Phlebotomus papatasi	43.84410376	33.63582349
Phlebotomus papatasi	43.40628294	34.59712573

species	lon	lat
Phlebotomus papatasi	46.21565	33.0434
Phlebotomus papatasi	47.36570605	33.69293055
Phlebotomus papatasi	48.10809788	34.1783406
Phlebotomus papatasi	48.64109715	33.57871642
Phlebotomus papatasi	49.06940013	34.34966179
Phlebotomus papatasi	50.03070238	33.50257367
Phlebotomus papatasi	49.7546849	32.75066399
Phlebotomus papatasi	49.05036444	32.12248629
Phlebotomus papatasi	49.74516706	31.96068294
Phlebotomus papatasi	49.74516706	31.18021973
Phlebotomus papatasi	49.01229307	31.27539817
Phlebotomus papatasi	49.12650719	30.58059555
Phlebotomus papatasi	50.90634403	32.23670041
Phlebotomus papatasi	52.13414591	32.80777106
Phlebotomus papatasi	50.99200462	34.09268
Phlebotomus papatasi	51.10621875	34.74941124
Phlebotomus papatasi	50.28768417	34.8065183
Phlebotomus papatasi	49.22168563	36.00576665
Phlebotomus papatasi	50.15443435	35.87251683
Phlebotomus papatasi	51.14429013	35.62505289
Phlebotomus papatasi	46.95643876	36.69105142
Phlebotomus papatasi	46.53765362	37.49055032
Phlebotomus papatasi	45.31936958	37.33826481
Phlebotomus papatasi	45.01479858	38.09969233
Phlebotomus papatasi	45.07190564	38.93726261
Phlebotomus papatasi	43.01605133	36.19612353
Phlebotomus papatasi	43.68230041	36.0819094
Phlebotomus papatasi	44.19626399	36.44358747
Phlebotomus papatasi	43.30158665	36.67201573
Phlebotomus papatasi	41.26476803	38.00451389
Phlebotomus papatasi	40.23684088	37.8331927
Phlebotomus papatasi	39.38023491	37.60476444
Phlebotomus papatasi	39.55155611	37.07176518
Phlebotomus papatasi	40.54141189	37.41440756
Phlebotomus papatasi	45.74648284	39.08954811
Phlebotomus papatasi	46.34134809	38.83375605
Phlebotomus papatasi	46.90052142	38.98247237
Phlebotomus papatasi	46.74585646	38.24483945
Phlebotomus papatasi	47.82851122	37.876023
Phlebotomus papatasi	48.56614413	39.14308598
Phlebotomus papatasi	47.40020823	39.38103208
Phlebotomus papatasi	47.05518639	39.85692429
Phlebotomus papatasi	47.780922	39.84502698
Phlebotomus papatasi	46.5055309	40.71115079
Phlebotomus papatasi	46.97190525	41.0157218
Phlebotomus papatasi	47.02901232	40.51127606

species	lon	lat
Phlebotomus papatasi	47.71429709	40.38754409
Phlebotomus papatasi	48.44717108	40.2162229
Phlebotomus papatasi	48.39958186	39.70225932
Phlebotomus papatasi	48.0802675	39.0393593
Phlebotomus papatasi	35.6255	37.362689
Phlebotomus papatasi	26.3323237	38.3233928
Phlebotomus papatasi	5.36978	43.296482
Phlebotomus papatasi	3.5787477	43.6975255
Phlebotomus papatasi	-1.1306544	37.9922399
Phlebotomus papatasi	-1.1328244	37.9419513
Phlebotomus papatasi	-2.467504	36.834775
Phlebotomus papatasi	-3.5985571	37.1773363
Phlebotomus papatasi	-3.7037902	40.4167754
Phlebotomus papatasi	-1.6760691	42.6953909
Phlebotomus papatasi	-7.449395	37.179432
Phlebotomus papatasi	-7.930834	37.0179538
Phlebotomus papatasi	4.2040933	36.6372932
Phlebotomus papatasi	19.089904	42.0912106
Phlebotomus papatasi	19.7431234	41.3715563
Phlebotomus papatasi	17.1204087	40.8544104
Phlebotomus pedifer	36.51012404	6.371526367
Phlebotomus pedifer	38.05677369	5.746917853
Phlebotomus pedifer	33.0137	3.8472
Phlebotomus pedifer	36.21269142	1.761320668
Phlebotomus pedifer	36.15320489	0.779793003
Phlebotomus pedifer	37.64036802	-0.082761612
Phlebotomus perfiliewi	48.0802675	39.0393593
Phlebotomus perfiliewi	35.6255	37.362689
Phlebotomus perfiliewi	4.2040933	36.6372932
Phlebotomus perfiliewi	19.7431234	41.3715563
Phlebotomus perfiliewi	19.6877385	41.8217526
Phlebotomus perfiliewi	17.1204087	40.8544104
Phlebotomus perfiliewi	-2.2209	34.968267
Phlebotomus perfiliewi	-0.598489574	35.334539
Phlebotomus perfiliewi	1.733382212	35.92940425
Phlebotomus perfiliewi	3.05475	36.6531
Phlebotomus perfiliewi	7.919980827	36.5242695
Phlebotomus perfiliewi	8.571953142	36.57661764
Phlebotomus perfiliewi	9.200130848	36.82408159
Phlebotomus perfiliewi	9.618915985	36.4814392
Phlebotomus perfiliewi	10.17095094	36.15783251
Phlebotomus perfiliewi	9.656987361	36.97636709
Phlebotomus perfiliewi	10.01866543	36.63372471
Phlebotomus perfiliewi	10.53262901	36.51951058
Phlebotomus perfiliewi	8.593963157	40.55507645
Phlebotomus perfiliewi	8.969917996	40.55031752

species	lon	lat
Phlebotomus perfiliewi	9.426774509	40.53604076
Phlebotomus perfiliewi	9.041301826	40.85012961
Phlebotomus perfiliewi	8.827150335	39.69371156
Phlebotomus perfiliewi	9.412497743	39.5937742
Phlebotomus perfiliewi	8.612998845	39.26540858
Phlebotomus perfiliewi	9.0685	39.1034
Phlebotomus perfiliewi	8.798596803	39.03698032
Phlebotomus perfiliewi	9.311	39.115967
Phlebotomus perfiliewi	13.42522078	37.7568303
Phlebotomus perfiliewi	14.09146986	37.52840205
Phlebotomus perfiliewi	14.89096876	37.83297306
Phlebotomus perfiliewi	14.8338617	37.07154553
Phlebotomus perfiliewi	21.00221778	39.85932205
Phlebotomus perfiliewi	21.06685	40.900467
Phlebotomus perfiliewi	20.71668246	41.47259661
Phlebotomus perfiliewi	21.65894902	41.40121278
Phlebotomus perfiliewi	20.6265	41.970033
Phlebotomus perfiliewi	21.70177932	41.94372989
Phlebotomus perfiliewi	21.701657	42.356421
Phlebotomus perfiliewi	20.78806629	42.45769347
Phlebotomus perfiliewi	20.87372688	43.07159441
Phlebotomus perfiliewi	21.50190459	43.05731764
Phlebotomus perfiliewi	21.67322578	43.58555798
Phlebotomus perfiliewi	22.64404587	43.72832564
Phlebotomus perfiliewi	23.61445	43.851033
Phlebotomus perfiliewi	20.47397744	43.57128122
Phlebotomus perfiliewi	21.18781574	43.89964684
Phlebotomus perfiliewi	21.83027021	44.27084275
Phlebotomus perfiliewi	19.90290679	44.05669126
Phlebotomus perfiliewi	20.67385216	44.37078012
Phlebotomus perfiliewi	21.2877531	44.95612752
Phlebotomus perfiliewi	19.98856739	44.68486897
Phlebotomus perfiliewi	20.63102186	45.24166284
Phlebotomus perfiliewi	19.88863003	45.29876991
Phlebotomus perfiliewi	18.91735	45.6071
Phlebotomus perfiliewi	20.0037	46.195833
Phlebotomus perfiliewi	19.75401202	45.63338161
Phlebotomus perfiliewi	23.69178204	35.41550016
Phlebotomus perfiliewi	24.14387963	35.37980825
Phlebotomus perfiliewi	23.02870557	40.86367271
Phlebotomus perfiliewi	23.66285	40.7273
Phlebotomus perfiliewi	23.3559	40.3133
Phlebotomus perfiliewi	25.17085	41.125533
Phlebotomus perfiliewi	28.07697004	45.1847739
Phlebotomus perfiliewi	28.4472	45.642067
Phlebotomus perfiliewi	28.68611206	44.97538133

species	lon	lat
Phlebotomus perfiliewi	29.18095	45.467533
Phlebotomus perfiliewi	34.5267767	45.39059727
Phlebotomus perfiliewi	33.54415	45.037667
Phlebotomus perfiliewi	33.4374	45.2689
Phlebotomus perfiliewi	33.72608808	44.6934152
Phlebotomus perfiliewi	34.18294459	44.80762933
Phlebotomus perfiliewi	34.48275668	44.96943268
Phlebotomus perfiliewi	34.75639469	45.0170219
Phlebotomus perfiliewi	33.66184263	44.87425424
Phlebotomus perfiliewi	35.05382732	45.11220034
Phlebotomus perfiliewi	35.33936264	45.18358417
Phlebotomus perfiliewi	35.62965688	45.23831177
Phlebotomus perfiliewi	35.94374573	45.25972692
Phlebotomus perfiliewi	36.23641944	45.30255722
Phlebotomus perfiliewi	37.58319437	45.06639571
Phlebotomus perfiliewi	15.8295	43.834
Phlebotomus perfiliewi	16.2419	43.6299
Phlebotomus perfiliewi	16.6426	43.522733
Phlebotomus perfiliewi	19.75222743	40.98284405
Phlebotomus perfiliewi	15.99648617	38.22266928
Phlebotomus perfiliewi	16.30105718	38.56531167
Phlebotomus perfiliewi	16.45334269	39.04120387
Phlebotomus perfiliewi	16.89116351	39.19348937
Phlebotomus perfiliewi	16.434307	39.55516745
Phlebotomus perfiliewi	16.33912856	39.97395258
Phlebotomus perfiliewi	16.32009287	40.48791616
Phlebotomus perfiliewi	16.66654239	40.497434
Phlebotomus perfiliewi	17.056774	40.70682657
Phlebotomus perfiliewi	17.55170189	40.57357676
Phlebotomus perfiliewi	17.87530858	40.48791616
Phlebotomus perfiliewi	18.21795097	40.19286299
Phlebotomus perfiliewi	15.2198301	40.32896816
Phlebotomus perfiliewi	15.2626604	40.88576204
Phlebotomus perfiliewi	15.69096338	41.52821651
Phlebotomus perfiliewi	15.3054907	41.34261855
Phlebotomus perfiliewi	14.87718772	41.34261855
Phlebotomus perfiliewi	14.94857155	41.71381447
Phlebotomus perfiliewi	14.27756354	41.07136
Phlebotomus perfiliewi	14.4203312	41.38544885
Phlebotomus perfiliewi	14.67731299	41.75664477
Phlebotomus perfiliewi	14.39177767	41.99934979
Phlebotomus perfiliewi	14.14907265	41.742368
Phlebotomus perfiliewi	13.8349838	41.51393975
Phlebotomus perfiliewi	13.34957375	41.65670741
Phlebotomus perfiliewi	13.67793937	41.97079626
Phlebotomus perfiliewi	13.99202822	42.31343864

species	lon	lat
Phlebotomus perfiliewi	12.83561017	41.6995377
Phlebotomus perfiliewi	12.906994	42.09928715
Phlebotomus perfiliewi	13.23535962	41.99934979
Phlebotomus perfiliewi	13.47806465	42.35626894
Phlebotomus perfiliewi	13.67793937	42.57042043
Phlebotomus perfiliewi	14.83435742	40.92859234
Phlebotomus perfiliewi	13.54944848	42.95589311
Phlebotomus perfiliewi	12.9498243	42.4990366
Phlebotomus perfiliewi	12.57862839	42.38482247
Phlebotomus perfiliewi	12.22170924	42.19922452
Phlebotomus perfiliewi	11.89334362	42.41337601
Phlebotomus perfiliewi	12.37875366	42.5846972
Phlebotomus perfiliewi	11.07956795	42.81312545
Phlebotomus perfiliewi	10.73692557	43.18432137
Phlebotomus perfiliewi	10.70837204	43.72683848
Phlebotomus perfiliewi	11.45076387	43.01300018
Phlebotomus perfiliewi	11.82195979	43.2128749
Phlebotomus perfiliewi	12.16460217	43.35564256
Phlebotomus perfiliewi	12.63573545	43.44130316
Phlebotomus perfiliewi	13.0354849	43.55551729
Phlebotomus perfiliewi	12.10749511	43.71256171
Phlebotomus perfiliewi	11.66491536	43.55551729
Phlebotomus perfiliewi	11.16522855	43.36991933
Phlebotomus perfiliewi	11.0767126	43.86960614
Phlebotomus perfiliewi	11.43363175	44.06948087
Phlebotomus perfiliewi	10.87683787	44.26935559
Phlebotomus perfiliewi	10.33432077	44.0552041
Phlebotomus perfiliewi	10.4057046	44.46923031
Phlebotomus perfiliewi	9.763250124	44.62627474
Phlebotomus perfiliewi	9.063688588	44.79759593
Phlebotomus perniciosus	0.34389	46.58472
Phlebotomus perniciosus	6.916178461	23.80467649
Phlebotomus perniciosus	7.748989813	23.80467649
Phlebotomus perniciosus	10.33719687	24.54876275
Phlebotomus perniciosus	-8.836448263	30.01744918
Phlebotomus perniciosus	-8.122609962	30.98113088
Phlebotomus perniciosus	-7.40877166	31.48081769
Phlebotomus perniciosus	-7.48015549	33.41294003
Phlebotomus perniciosus	-6.692553897	34.06967127
Phlebotomus perniciosus	-5.978715595	34.86679071
Phlebotomus perniciosus	-5.87163985	34.21243893
Phlebotomus perniciosus	-4.943650058	34.29572006
Phlebotomus perniciosus	-5.502823394	33.68895751
Phlebotomus perniciosus	-6.252353611	33.49860063
Phlebotomus perniciosus	-4.872266228	32.90373538
Phlebotomus perniciosus	-2.980594728	34.86679071

species	lon	lat
Phlebotomus perniciosus	-1.65999387	34.91437993
Phlebotomus perniciosus	-0.886669043	35.0928395
Phlebotomus perniciosus	-1.350663939	34.31951467
Phlebotomus perniciosus	0.65760115	33.6770602
Phlebotomus perniciosus	2.52547804	35.74719128
Phlebotomus perniciosus	2.751526835	36.24687809
Phlebotomus perniciosus	5.095295926	36.44913227
Phlebotomus perniciosus	5.892415363	36.42533766
Phlebotomus perniciosus	5.345139331	34.49797425
Phlebotomus perniciosus	6.082772243	34.42659042
Phlebotomus perniciosus	7.071438291	35.98513738
Phlebotomus perniciosus	7.844763118	36.0327266
Phlebotomus perniciosus	8.65377986	34.03397935
Phlebotomus perniciosus	8.820342131	34.58125538
Phlebotomus perniciosus	8.844136741	35.22370986
Phlebotomus perniciosus	9.605564262	35.37837482
Phlebotomus perniciosus	8.998801706	35.83047241
Phlebotomus perniciosus	7.691287883	36.69897568
Phlebotomus perniciosus	8.315150608	36.68593984
Phlebotomus perniciosus	8.788219407	36.70611406
Phlebotomus perniciosus	9.130861792	36.79891304
Phlebotomus perniciosus	9.437812262	36.90598879
Phlebotomus perniciosus	9.830423327	37.00592615
Phlebotomus perniciosus	9.644825369	36.22070402
Phlebotomus perniciosus	10.0945435	36.04938283
Phlebotomus perniciosus	10.3872172	35.81381619
Phlebotomus perniciosus	9.801869795	36.45627066
Phlebotomus perniciosus	10.70130606	36.62759185
Phlebotomus perniciosus	10.19448086	36.34205653
Phlebotomus perniciosus	-6.720512564	37.35154286
Phlebotomus perniciosus	-5.982879652	36.80426683
Phlebotomus perniciosus	-5.649755111	36.39975846
Phlebotomus perniciosus	-7.577118526	38.68404102
Phlebotomus perniciosus	-6.601539514	38.66024641
Phlebotomus perniciosus	-6.220825753	39.23131706
Phlebotomus perniciosus	-6.92155	40.213267
Phlebotomus perniciosus	-5.840111992	40.18310146
Phlebotomus perniciosus	-7.577118526	41.44421579
Phlebotomus perniciosus	-7.362967035	40.73037749
Phlebotomus perniciosus	-6.88605	40.718667
Phlebotomus perniciosus	-5.959085042	40.94452898
Phlebotomus perniciosus	-6.101852702	41.56318884
Phlebotomus perniciosus	-5.17386291	41.65836728
Phlebotomus perniciosus	-5.17386291	41.03970742
Phlebotomus perniciosus	-4.198283898	41.68216189
Phlebotomus perniciosus	-3.603418646	41.61077806

species	lon	lat
Phlebotomus perniciosus	-5.17386291	41.03970742
Phlebotomus perniciosus	-4.412435388	40.96832359
Phlebotomus perniciosus	-3.008553395	42.22943792
Phlebotomus perniciosus	-2.247125873	42.22943792
Phlebotomus perniciosus	-2.223331263	41.63457267
Phlebotomus perniciosus	-3.103731835	40.80176132
Phlebotomus perniciosus	-4.293462338	40.08792302
Phlebotomus perniciosus	-3.389267156	39.96894997
Phlebotomus perniciosus	-2.723018074	40.13551224
Phlebotomus perniciosus	-4.893086511	39.29794196
Phlebotomus perniciosus	-4.122141145	39.39787933
Phlebotomus perniciosus	-3.308365481	39.38360256
Phlebotomus perniciosus	-3.037106927	38.95529958
Phlebotomus perniciosus	-2.037733304	38.82680868
Phlebotomus perniciosus	-1.966349474	38.14152391
Phlebotomus perniciosus	-1.381002067	39.61203082
Phlebotomus perniciosus	-0.767101127	38.92674605
Phlebotomus perniciosus	-0.738547595	38.3985057
Phlebotomus perniciosus	-1.281064704	37.65611387
Phlebotomus perniciosus	-5.07868447	36.91372204
Phlebotomus perniciosus	-4.835979447	37.39913208
Phlebotomus perniciosus	-4.25063204	37.0136594
Phlebotomus perniciosus	-3.765221995	37.07076646
Phlebotomus perniciosus	-3.308365481	37.02793616
Phlebotomus perniciosus	-2.308991859	37.0564897
Phlebotomus perniciosus	-2.865785734	37.04221293
Phlebotomus perniciosus	-5.07868447	38.04158655
Phlebotomus perniciosus	-4.364846168	37.88454213
Phlebotomus perniciosus	-3.836605825	37.42768561
Phlebotomus perniciosus	-3.965096719	38.24146128
Phlebotomus perniciosus	-3.42257961	37.94164919
Phlebotomus perniciosus	-2.865785734	37.61328357
Phlebotomus perniciosus	0.223706436	40.42580648
Phlebotomus perniciosus	1.05935	40.968133
Phlebotomus perniciosus	2.22245368	41.53939423
Phlebotomus perniciosus	1.979748658	41.93914368
Phlebotomus perniciosus	0.380750862	41.73926896
Phlebotomus perniciosus	0.433099004	43.17170448
Phlebotomus perniciosus	1.1064	42.735033
Phlebotomus perniciosus	1.498511	42.614993
Phlebotomus perniciosus	1.91431348	42.61598136
Phlebotomus perniciosus	2.22475	42.3865
Phlebotomus perniciosus	2.785196208	42.05918749
Phlebotomus perniciosus	2.628151782	42.5588743
Phlebotomus perniciosus	2.399723525	42.87296315
Phlebotomus perniciosus	3.027901231	43.14422171

species	lon	lat
Phlebotomus perniciosus	1.84292965	43.45831056
Phlebotomus perniciosus	1.557394329	44.15787209
Phlebotomus perniciosus	1.171921646	44.80032657
Phlebotomus perniciosus	0.18682479	43.97227414
Phlebotomus perniciosus	-0.070156999	44.7003892
Phlebotomus perniciosus	-0.912486195	45.91391432
Phlebotomus perniciosus	-0.702498761	45.36485369
Phlebotomus perniciosus	-0.309887695	45.25777794
Phlebotomus perniciosus	0.052980108	45.33511043
Phlebotomus perniciosus	0.463437132	45.53736461
Phlebotomus perniciosus	1.135634866	46.08583037
Phlebotomus perniciosus	1.064251036	46.49985659
Phlebotomus perniciosus	1.04997427	47.0423737
Phlebotomus perniciosus	-1.162924466	47.32790902
Phlebotomus perniciosus	-0.806005315	48.42722
Phlebotomus perniciosus	-0.306318503	47.756212
Phlebotomus perniciosus	0.307582436	48.25589881
Phlebotomus perniciosus	0.250475372	47.78476553
Phlebotomus perniciosus	1.720982274	48.6413715
Phlebotomus perniciosus	2.063624658	49.42659363
Phlebotomus perniciosus	2.363436745	48.75558562
Phlebotomus perniciosus	2.648972066	49.32665626
Phlebotomus perniciosus	2.520481171	47.91325643
Phlebotomus perniciosus	2.791739726	48.61281796
Phlebotomus perniciosus	3.134382111	48.89835328
Phlebotomus perniciosus	3.67689922	48.5557109
Phlebotomus perniciosus	4.148032499	48.84124622
Phlebotomus perniciosus	2.09217819	46.69973131
Phlebotomus perniciosus	3.819666881	46.22859804
Phlebotomus perniciosus	3.291426537	45.60042033
Phlebotomus perniciosus	4.062371903	45.58614356
Phlebotomus perniciosus	5.247343484	46.2000445
Phlebotomus perniciosus	4.804763737	45.62897386
Phlebotomus perniciosus	5.547155571	45.60042033
Phlebotomus perniciosus	5.161682888	48.56998766
Phlebotomus perniciosus	5.161682888	47.8418726
Phlebotomus perniciosus	3.23574715	43.73016398
Phlebotomus perniciosus	3.863924855	44.55821641
Phlebotomus perniciosus	5.291601459	44.84375173
Phlebotomus perniciosus	3.9637	43.528533
Phlebotomus perniciosus	4.320781368	44.0156993
Phlebotomus perniciosus	4.791914648	44.35834168
Phlebotomus perniciosus	4.49535	43.381
Phlebotomus perniciosus	4.649146987	43.75871751
Phlebotomus perniciosus	5.063173202	44.07280636
Phlebotomus perniciosus	4.9528	43.3079

species	lon	lat
Phlebotomus perniciosus	5.134557032	43.68733368
Phlebotomus perniciosus	5.57713678	44.0156993
Phlebotomus perniciosus	5.5119	43.1305
Phlebotomus perniciosus	5.777011504	43.60167308
Phlebotomus perniciosus	6.119653889	43.27330747
Phlebotomus perniciosus	6.633617466	43.54456602
Phlebotomus perniciosus	7.047643681	43.71588721
Phlebotomus perniciosus	7.53715	43.926867
Phlebotomus perniciosus	8.018463772	44.22985079
Phlebotomus perniciosus	8.032740538	44.58676994
Phlebotomus perniciosus	7.44739313	44.34406492
Phlebotomus perniciosus	6.933429553	44.0156993
Phlebotomus perniciosus	6.448019508	43.98714577
Phlebotomus perniciosus	8.803685903	46.38564246
Phlebotomus perniciosus	9.476121584	44.40117198
Phlebotomus perniciosus	9.733103372	44.94368909
Phlebotomus perniciosus	8.69585	44.3602
Phlebotomus perniciosus	10.00436193	45.14356382
Phlebotomus perniciosus	10.46121844	44.95796586
Phlebotomus perniciosus	11.11794968	44.80092143
Phlebotomus perniciosus	11.50818128	45.32083366
Phlebotomus perniciosus	12.07325	45.304767
Phlebotomus perniciosus	12.41059187	45.86573023
Phlebotomus perniciosus	13.28535	45.755167
Phlebotomus perniciosus	13.77465	45.547667
Phlebotomus perniciosus	14.115177	45.387334
Phlebotomus perniciosus	13.83826847	45.18044546
Phlebotomus perniciosus	14.115177	45.387334
Phlebotomus perniciosus	14.55845	45.246133
Phlebotomus perniciosus	14.91515	44.937467
Phlebotomus perniciosus	15.07945	44.589133
Phlebotomus perniciosus	9.084105383	42.42502962
Phlebotomus perniciosus	8.998444787	41.56842366
Phlebotomus perniciosus	8.612972104	40.59760356
Phlebotomus perniciosus	9.141212447	40.72609446
Phlebotomus perniciosus	8.84140036	40.32634501
Phlebotomus perniciosus	9.269703341	40.4976662
Phlebotomus perniciosus	9.255426575	40.01225616
Phlebotomus perniciosus	8.56535	39.658567
Phlebotomus perniciosus	8.955614489	39.35552492
Phlebotomus perniciosus	12.73895749	37.88501802
Phlebotomus perniciosus	13.01995	38.075
Phlebotomus perniciosus	13.16726047	37.71369683
Phlebotomus perniciosus	13.49562609	37.94212508
Phlebotomus perniciosus	13.53585	37.2759
Phlebotomus perniciosus	13.49562609	37.94212508

species	lon	lat
Phlebotomus perniciosus	13.78116141	37.77080389
Phlebotomus perniciosus	13.62411698	37.58520593
Phlebotomus perniciosus	13.98103613	37.38533121
Phlebotomus perniciosus	14.0809735	37.72797359
Phlebotomus perniciosus	14.12380379	37.34250091
Phlebotomus perniciosus	14.49499971	37.79935742
Phlebotomus perniciosus	14.90902593	37.79935742
Phlebotomus perniciosus	14.30940175	37.41388474
Phlebotomus perniciosus	14.7948118	37.38533121
Phlebotomus perniciosus	15.2347	36.9525
Phlebotomus perniciosus	14.2855	37.0767
Phlebotomus perniciosus	13.9164	37.1142
Phlebotomus perniciosus	14.65204414	37.0807602
Phlebotomus perniciosus	15.92600632	38.48963084
Phlebotomus perniciosus	16.2467589	38.34901291
Phlebotomus perniciosus	16.40142387	38.88439164
Phlebotomus perniciosus	16.66375	38.8255
Phlebotomus perniciosus	16.30624543	39.2532081
Phlebotomus perniciosus	16.29434812	38.59885632
Phlebotomus perniciosus	15.91363436	38.12296412
Phlebotomus perniciosus	15.91363436	38.12296412
Phlebotomus perniciosus	16.78213763	39.19372157
Phlebotomus perniciosus	16.86541876	39.40787306
Phlebotomus perniciosus	16.53229422	39.36028384
Phlebotomus perniciosus	16.99628912	39.11044044
Phlebotomus perniciosus	16.43711578	39.64581916
Phlebotomus perniciosus	16.13968316	39.51494881
Phlebotomus perniciosus	15.98501819	39.75289491
Phlebotomus perniciosus	15.85414784	40.13360867
Phlebotomus perniciosus	16.4728077	39.9789437
Phlebotomus perniciosus	16.63936997	40.43104129
Phlebotomus perniciosus	16.77024032	40.71657662
Phlebotomus perniciosus	16.80593224	41.01400924
Phlebotomus perniciosus	17.19854331	40.78796045
Phlebotomus perniciosus	16.99628912	40.71657662
Phlebotomus perniciosus	17.04387834	40.99021463
Phlebotomus perniciosus	17.37700288	40.52621973
Phlebotomus perniciosus	17.43648941	40.74037123
Phlebotomus perniciosus	17.59115437	40.51432243
Phlebotomus perniciosus	17.7294129	40.78538523
Phlebotomus perniciosus	17.85289508	40.4548359
Phlebotomus perniciosus	18.12445	40.6009
Phlebotomus perniciosus	18.18601962	40.19309519
Phlebotomus perniciosus	18.33695	40.419733
Phlebotomus perniciosus	15.47343408	40.25258172
Phlebotomus perniciosus	15.15220684	40.49052782

species	lon	lat
Phlebotomus perniciosus	15.59240713	40.70467931
Phlebotomus perniciosus	15.86366568	41.28764726
Phlebotomus perniciosus	15.62096066	41.730227
Phlebotomus perniciosus	15.89697813	41.8158876
Phlebotomus perniciosus	15.19694071	40.90883706
Phlebotomus perniciosus	14.64014683	40.90883706
Phlebotomus perniciosus	14.98278922	41.35141681
Phlebotomus perniciosus	14.16901355	41.13726532
Phlebotomus perniciosus	14.72580743	41.87965715
Phlebotomus perniciosus	14.36888828	41.85110362
Phlebotomus perniciosus	13.91203176	41.63695213
Phlebotomus perniciosus	13.57585	41.267233
Phlebotomus perniciosus	14.64014683	41.28003298
Phlebotomus perniciosus	14.48310241	41.50846124
Phlebotomus perniciosus	14.48310241	41.50846124
Phlebotomus perniciosus	9.828876678	44.20677002
Phlebotomus perniciosus	10.30000996	44.06400236
Phlebotomus perniciosus	10.97101796	44.09255589
Phlebotomus perniciosus	11.51353507	44.22104678
Phlebotomus perniciosus	12.01322188	44.56368917
Phlebotomus perniciosus	10.52843821	43.40727112
Phlebotomus perniciosus	11.44215124	43.66425291
Phlebotomus perniciosus	12.11315924	43.8784044
Phlebotomus perniciosus	10.9139109	42.90758431
Phlebotomus perniciosus	12.58429252	43.62142261
Phlebotomus perniciosus	13.35523789	43.40727112
Phlebotomus perniciosus	11.87045422	43.32161052
Phlebotomus perniciosus	12.75561372	43.02179844
Phlebotomus perniciosus	12.32731073	43.17884286
Phlebotomus perniciosus	11.61105	42.2109
Phlebotomus perniciosus	11.94585	41.8942
Phlebotomus perniciosus	13.46945202	42.03670158
Phlebotomus perniciosus	14.09762972	42.37934397
Phlebotomus perniciosus	13.16963993	41.39424711
Phlebotomus perniciosus	13.65504998	42.99324491
Phlebotomus perniciosus	13.08397933	42.73626312
Phlebotomus perniciosus	12.22737337	42.69343282
Phlebotomus perniciosus	12.52718546	42.60777222
Phlebotomus perniciosus	7.7730563	49.5756828
Phlebotomus perniciosus	9.2041538	42.4651109
Phlebotomus perniciosus	5.927999	43.124228
Phlebotomus perniciosus	5.36978	43.296482
Phlebotomus perniciosus	6.76837	43.42519
Phlebotomus perniciosus	7.2619532	43.7101728
Phlebotomus perniciosus	5.3923682	43.2846279
Phlebotomus perniciosus	3.5787477	43.6975255

species	lon	lat
Phlebotomus perniciosus	0.68484	47.394144
Phlebotomus perniciosus	-1.1306544	37.9922399
Phlebotomus perniciosus	-1.1328244	37.9419513
Phlebotomus perniciosus	-2.467504	36.834775
Phlebotomus perniciosus	-3.5985571	37.1773363
Phlebotomus perniciosus	-4.1104449	36.840268
Phlebotomus perniciosus	-5.6635397	40.9701039
Phlebotomus perniciosus	-3.7037902	40.4167754
Phlebotomus perniciosus	0.810952	41.2133495
Phlebotomus perniciosus	0.8454749	41.2344554
Phlebotomus perniciosus	2.1734035	41.3850639
Phlebotomus perniciosus	1.5208624	41.5911589
Phlebotomus perniciosus	-1.6760691	42.6953909
Phlebotomus perniciosus	-16.6291304	28.2915637
Phlebotomus perniciosus	-7.7524102	41.6214901
Phlebotomus perniciosus	-7.747452	41.299773
Phlebotomus perniciosus	-7.9093074	38.5707119
Phlebotomus perniciosus	-7.449395	37.179432
Phlebotomus perniciosus	-9.1393366	38.7222524
Phlebotomus perniciosus	-8.6406106	41.1514848
Phlebotomus perniciosus	-7.930834	37.0179538
Phlebotomus perniciosus	-8.5335055	37.1417854
Phlebotomus perniciosus	-9.2226193	30.0068629
Phlebotomus perniciosus	-7.9085632	31.4081287
Phlebotomus perniciosus	-5.3677844	33.685735
Phlebotomus perniciosus	-7.6758373	31.2258103
Phlebotomus perniciosus	-7.6816885	31.2655479
Phlebotomus perniciosus	-6.0017674	33.425066
Phlebotomus perniciosus	-8.3892999	31.3597821
Phlebotomus perniciosus	-7.782511	31.374709
Phlebotomus perniciosus	-6.979304	31.8462611
Phlebotomus perniciosus	-9.0832365	31.2174305
Phlebotomus perniciosus	-5.8432964	33.1584786
Phlebotomus perniciosus	-5.0880863	31.1488978
Phlebotomus perniciosus	-5.567558	34.7953732
Phlebotomus perniciosus	14.2512221	36.0442999
Phlebotomus perniciosus	15.75	41.733
Phlebotomus perniciosus	7.2619532	43.7101728
Phlebotomus perniciosus	37.1773363	4037
Phlebotomus perniciosus	-7.747452	41.299773
Phlebotomus perniciosus	4.2040933	36.6372932
Phlebotomus perniciosus	-7.6310791	31.5438441
Phlebotomus perniciosus	-7.4841222	31.5319623
Phlebotomus perniciosus	-7.6758373	31.2258103
Phlebotomus perniciosus	-7.9504327	31.3526424
Phlebotomus perniciosus	-9.4799447	30.4854645

species	lon	lat
Phlebotomus perniciosus	-9.6584978	30.8661606
Phlebotomus perniciosus	-7.0434589	32.0772052
Phlebotomus perniciosus	-9.6035176	30.4751364
Phlebotomus perniciosus	-5.661571	32.9340471
Phlebotomus perniciosus	-6.5730004	31.9649107
Phlebotomus perniciosus	-8.0751645	30.6262829
Phlebotomus perniciosus	10.3385624	33.0641739
Phlebotomus perniciosus	17.1204087	40.8544104
Phlebotomus rodhaini	-1.65	37.7
Phlebotomus rodhaini	-16.26018508	14.93425756
Phlebotomus rodhaini	-15.83665	13.179467
Phlebotomus rodhaini	-11.89875	12.5608
Phlebotomus rodhaini	-9.988322446	12.38823429
Phlebotomus rodhaini	-7.787321015	12.5369506
Phlebotomus rodhaini	-10.28575507	9.830313706
Phlebotomus rodhaini	0.986941444	8.938015829
Phlebotomus rodhaini	6.84636417	12.18003145
Phlebotomus rodhaini	11.04016419	5.993432834
Phlebotomus rodhaini	36.49315	14.922533
Phlebotomus rodhaini	37.76745994	12.89882696
Phlebotomus rodhaini	36.96439185	11.59012341
Phlebotomus rodhaini	34.52544432	12.98805675
Phlebotomus rodhaini	33.90083581	11.38192057
Phlebotomus rodhaini	33.78186276	10.37064964
Phlebotomus rodhaini	32.4731592	9.746041129
Phlebotomus rodhaini	31.2834287	7.753242536
Phlebotomus rodhaini	37.76745994	7.188120547
Phlebotomus rodhaini	28.4895449	5.841742195
Phlebotomus rodhaini	26.01490546	5.746563755
Phlebotomus rodhaini	27.79995	4.691033
Phlebotomus rodhaini	28.62636391	3.991711263
Phlebotomus rodhaini	30.29198661	4.705549565
Phlebotomus rodhaini	31.03556818	3.961968
Phlebotomus rodhaini	29.60789158	3.069670123
Phlebotomus rodhaini	29.51866179	1.790709832
Phlebotomus rodhaini	32.04683911	2.742494235
Phlebotomus rodhaini	32.82016393	1.195844581
Phlebotomus rodhaini	34.4504	0.9494
Phlebotomus rodhaini	36.06217955	0.600979329
Phlebotomus rodhaini	38.11446467	-0.529264648
Phlebotomus rodhaini	38.14420793	-1.778481676
Phlebotomus rodhaini	39.3636817	-3.473847643
Phlebotomus rodhaini	27.16894404	0.660465855
Phlebotomus rodhaini	26.63356532	-1.064643375
Phlebotomus rodhaini	19.83925	5.116667
Phlebotomus rodhaini	15.45009859	0.928155218

species	lon	lat
Phlebotomus rodhaini	16.37213973	-0.886183799
Phlebotomus rodhaini	15.71778795	-3.592820693
Phlebotomus rodhaini	14.79574681	-4.157942682
Phlebotomus rodhaini	12.86243475	-3.295388068
Phlebotomus rodhaini	21.13106174	-8.084053342
Phlebotomus rodhaini	21.50681829	-17.21523495
Phlebotomus rodhaini	16.84307472	-19.4519283
Phlebotomus rodhaini	28.97832585	-20.11817738
Phlebotomus rodhaini	29.97769947	-21.26031866
Phlebotomus rodhaini	31.02466232	-23.59219045
Phlebotomus rodhaini	31.6026	-22.971967
Phlebotomus saevus	-0.77519	36.34763
Phlebotomus salehi	50.34915105	30.89103036
Phlebotomus salehi	56.82128499	27.70255261
Phlebotomus salehi	57.10682031	29.08264
Phlebotomus salehi	57.86824783	27.36942807
Phlebotomus salehi	62.19886686	27.13148197
Phlebotomus salehi	62.34163452	26.08451913
Phlebotomus salehi	72.66849529	28.36880169
Phlebotomus salehi	74.00099345	29.27299688
Phlebotomus sergenti	-10.13202986	28.19889547
Phlebotomus sergenti	-9.953570285	29.01980952
Phlebotomus sergenti	-9.846018647	29.80868016
Phlebotomus sergenti	-9.122662502	30.10373333
Phlebotomus sergenti	-9.113144658	30.84612516
Phlebotomus sergenti	-8.494484796	28.98062773
Phlebotomus sergenti	-7.666432366	29.56121622
Phlebotomus sergenti	-6.989872287	29.94113682
Phlebotomus sergenti	-6.323623205	29.92210114
Phlebotomus sergenti	-6.589	29.503167
Phlebotomus sergenti	-8.722119899	32.2063837
Phlebotomus sergenti	-7.722746277	32.51095471
Phlebotomus sergenti	-8.760191275	31.51158109
Phlebotomus sergenti	-8.160567102	31.60675953
Phlebotomus sergenti	-7.713228433	31.24508145
Phlebotomus sergenti	-7.741781965	30.76918925
Phlebotomus sergenti	-7.522871552	31.8161521
Phlebotomus sergenti	-6.837586782	31.63531306
Phlebotomus sergenti	-5.562195683	32.07313388
Phlebotomus sergenti	-5.038714262	31.79711641
Phlebotomus sergenti	-5.590749215	31.30218852
Phlebotomus sergenti	-5.723999032	30.65497512
Phlebotomus sergenti	-5.666891968	30.02679742
Phlebotomus sergenti	-4.705589721	30.84533201
Phlebotomus sergenti	-4.819803849	30.20763646
Phlebotomus sergenti	-4.210661832	29.9792082

species	lon	lat
Phlebotomus sergenti	-7.027943663	33.17720379
Phlebotomus sergenti	-6.599640682	32.85359709
Phlebotomus sergenti	-5.847731004	33.03443613
Phlebotomus sergenti	-6.599640682	33.42466774
Phlebotomus sergenti	-6.02857004	33.79586365
Phlebotomus sergenti	-5.409910179	34.00525622
Phlebotomus sergenti	-5.029196418	33.37707852
Phlebotomus sergenti	-4.439090089	33.85297072
Phlebotomus sergenti	-5.942909444	34.42404136
Phlebotomus sergenti	-4.817126956	32.6028019
Phlebotomus sergenti	-4.11756542	32.67418573
Phlebotomus sergenti	-3.460834183	32.46003424
Phlebotomus sergenti	-3.503664481	31.87468684
Phlebotomus sergenti	-2.775549413	31.43210709
Phlebotomus sergenti	-2.475737326	32.65990897
Phlebotomus sergenti	-1.1251	32.457
Phlebotomus sergenti	0.722258266	34.18752293
Phlebotomus sergenti	-0.691141572	34.80142387
Phlebotomus sergenti	-2.304416134	34.67293298
Phlebotomus sergenti	-2.904040307	34.2017997
Phlebotomus sergenti	-1.6494	34.413033
Phlebotomus sergenti	-2.233032304	33.91626438
Phlebotomus sergenti	-3.28951299	33.73066642
Phlebotomus sergenti	-3.246682692	33.13104225
Phlebotomus sergenti	-7.016	37.161433
Phlebotomus sergenti	-5.716563216	37.21419733
Phlebotomus sergenti	-5.759393514	36.25765401
Phlebotomus sergenti	-4.631528997	37.28558116
Phlebotomus sergenti	-4.53755	36.6202
Phlebotomus sergenti	-3.5979	36.643133
Phlebotomus sergenti	-2.589951454	37.09998321
Phlebotomus sergenti	-1.362149575	37.68533061
Phlebotomus sergenti	-1.019507191	38.47055274
Phlebotomus sergenti	-2.047434345	38.22784772
Phlebotomus sergenti	-3.503664481	37.57111648
Phlebotomus sergenti	-3.403727118	38.29923155
Phlebotomus sergenti	-4.360270443	38.41344568
Phlebotomus sergenti	-4.403100741	39.24149811
Phlebotomus sergenti	-5.573795556	39.11300722
Phlebotomus sergenti	-6.53033888	39.28432841
Phlebotomus sergenti	-6.658829774	40.22659497
Phlebotomus sergenti	-5.331090533	40.15521114
Phlebotomus sergenti	-4.203226016	40.24087173
Phlebotomus sergenti	-3.175298862	40.1694879
Phlebotomus sergenti	-5.602349088	41.08320093
Phlebotomus sergenti	-6.630276242	40.98326357

species	lon	lat
Phlebotomus sergenti	-7.529712502	40.94043327
Phlebotomus sergenti	-8.172166974	41.46867361
Phlebotomus sergenti	-6.430401518	42.16823515
Phlebotomus sergenti	-5.573795556	42.02546749
Phlebotomus sergenti	-8.959530621	38.70258985
Phlebotomus sergenti	3.19983238	36.47128995
Phlebotomus sergenti	3.05706472	35.82883548
Phlebotomus sergenti	3.114171784	33.15908023
Phlebotomus sergenti	5.055811965	35.92877284
Phlebotomus sergenti	6.968898614	36.35707582
Phlebotomus sergenti	7.12594304	35.28631837
Phlebotomus sergenti	6.22650678	31.95983188
Phlebotomus sergenti	8.111039896	33.77298117
Phlebotomus sergenti	8.539342877	33.45889232
Phlebotomus sergenti	9.310288243	33.38750849
Phlebotomus sergenti	8.867708496	35.87166578
Phlebotomus sergenti	8.068209598	36.55695055
Phlebotomus sergenti	8.85343173	36.71399497
Phlebotomus sergenti	9.795698289	36.58550408
Phlebotomus sergenti	10.48098306	32.74505401
Phlebotomus sergenti	12.06570409	32.2453672
Phlebotomus sergenti	10.1084	31.381667
Phlebotomus sergenti	9.221653321	29.0688859
Phlebotomus sergenti	8.6981719	24.51221808
Phlebotomus sergenti	7.080138416	24.30996389
Phlebotomus sergenti	7.258597991	23.72699595
Phlebotomus sergenti	7.282392601	22.76331424
Phlebotomus sergenti	7.437057567	21.91860558
Phlebotomus sergenti	8.857595787	20.54089766
Phlebotomus sergenti	13.95059412	37.378281
Phlebotomus sergenti	14.71202165	37.60670926
Phlebotomus sergenti	14.73105733	37.01660293
Phlebotomus sergenti	22.34104952	37.2926204
Phlebotomus sergenti	14.60383549	40.86617426
Phlebotomus sergenti	38.4451222	11.86205195
Phlebotomus sergenti	37.63610546	9.994175063
Phlebotomus sergenti	39.27793355	10.48196457
Phlebotomus sergenti	46.15575	10.718567
Phlebotomus sergenti	43.75052709	14.91886619
Phlebotomus sergenti	44.58809737	14.34779555
Phlebotomus sergenti	43.90265397	13.3787204
Phlebotomus sergenti	44.42613539	13.48579614
Phlebotomus sergenti	46.97334839	24.63476069
Phlebotomus sergenti	39.03546648	25.37715252
Phlebotomus sergenti	31.7989	36.634967
Phlebotomus sergenti	34.39789698	37.2958727

species	lon	lat
Phlebotomus sergenti	34.27805	36.6962
Phlebotomus sergenti	33.4597449	35.10999076
Phlebotomus sergenti	32.65072816	34.84825005
Phlebotomus sergenti	31.63707777	30.36588089
Phlebotomus sergenti	34.985505	31.963992
Phlebotomus sergenti	35.80113453	33.97076432
Phlebotomus sergenti	37.02655695	36.31453341
Phlebotomus sergenti	43.726673	42.72390906
Phlebotomus sergenti	44.65942171	42.69535553
Phlebotomus sergenti	45.12579607	42.22898117
Phlebotomus sergenti	46.12516969	41.26767892
Phlebotomus sergenti	46.7294	41.2811
Phlebotomus sergenti	47.64065	41.2846
Phlebotomus sergenti	46.6676868	40.72516181
Phlebotomus sergenti	46.79141877	40.14457333
Phlebotomus sergenti	44.6784574	40.38251943
Phlebotomus sergenti	45.05917116	39.9446986
Phlebotomus sergenti	46.066	39.1224
Phlebotomus sergenti	44.79267153	38.87870007
Phlebotomus sergenti	45.21145666	37.40343425
Phlebotomus sergenti	44.04076185	36.87043498
Phlebotomus sergenti	44.55472543	36.53731044
Phlebotomus sergenti	44.4405113	35.95672196
Phlebotomus sergenti	43.77426222	36.36598925
Phlebotomus sergenti	43.15455	37.436767
Phlebotomus sergenti	43.03187038	36.71814948
Phlebotomus sergenti	43.19367373	36.06141824
Phlebotomus sergenti	42.42272836	36.26129297
Phlebotomus sergenti	46.38254805	42.15135125
Phlebotomus sergenti	46.95395	41.6479
Phlebotomus sergenti	48.52691831	40.78078172
Phlebotomus sergenti	49.19792632	40.52379993
Phlebotomus sergenti	49.82610402	40.49524639
Phlebotomus sergenti	48.73393142	39.21033745
Phlebotomus sergenti	48.60945562	38.50678708
Phlebotomus sergenti	47.88589152	37.38219756
Phlebotomus sergenti	48.69966718	37.56779552
Phlebotomus sergenti	50.21300438	36.61125219
Phlebotomus sergenti	51.16954771	35.55477151
Phlebotomus sergenti	50.14162055	35.12646853
Phlebotomus sergenti	50.64130736	34.39835346
Phlebotomus sergenti	51.58357392	33.81300605
Phlebotomus sergenti	52.69716167	33.89866665
Phlebotomus sergenti	42.14663157	34.35552316
Phlebotomus sergenti	45.10192214	34.08426461
Phlebotomus sergenti	44.78069491	33.39612448

species	lon	lat
Phlebotomus sergenti	45.43028776	33.44609316
Phlebotomus sergenti	44.03830307	33.14628108
Phlebotomus sergenti	44.28100809	32.68228618
Phlebotomus sergenti	44.84494035	32.86074576
Phlebotomus sergenti	43.63141524	32.21829129
Phlebotomus sergenti	44.1953475	32.14690746
Phlebotomus sergenti	44.72358784	32.12549231
Phlebotomus sergenti	44.52371312	31.5401449
Phlebotomus sergenti	46.64381287	30.71923085
Phlebotomus sergenti	48.2713642	30.83344498
Phlebotomus sergenti	48.88526514	31.79712669
Phlebotomus sergenti	47.82164607	32.99637503
Phlebotomus sergenti	46.79371892	33.90294968
Phlebotomus sergenti	47.77167739	33.92436483
Phlebotomus sergenti	48.61400659	33.83156585
Phlebotomus sergenti	50.75760352	31.73421969
Phlebotomus sergenti	53.29172949	32.75738792
Phlebotomus sergenti	51.69749062	29.1525045
Phlebotomus sergenti	52.93481034	29.94962393
Phlebotomus sergenti	54.54094652	30.49689996
Phlebotomus sergenti	55.75447163	32.38857146
Phlebotomus sergenti	54.45766538	29.44993712
Phlebotomus sergenti	55.9091366	29.29527216
Phlebotomus sergenti	57.71752696	30.50879727
Phlebotomus sergenti	59.53781463	30.79433259
Phlebotomus sergenti	58.32428952	29.48562904
Phlebotomus sergenti	59.13330626	29.77116436
Phlebotomus sergenti	59.79955534	29.93772663
Phlebotomus sergenti	59.4545335	28.90266109
Phlebotomus sergenti	60.32303676	32.79307983
Phlebotomus sergenti	58.46705718	34.05419417
Phlebotomus sergenti	60.08509066	34.99408127
Phlebotomus sergenti	55.26668213	35.26771928
Phlebotomus sergenti	55.69498511	36.05294141
Phlebotomus sergenti	56.75384525	36.9809312
Phlebotomus sergenti	57.39629973	36.08863333
Phlebotomus sergenti	57.99116498	37.13559617
Phlebotomus sergenti	58.95484668	36.46934709
Phlebotomus sergenti	59.62109577	37.05231504
Phlebotomus sergenti	60.01370683	37.77805064
Phlebotomus sergenti	59.89473378	38.33722398
Phlebotomus sergenti	59.26417662	38.1587644
Phlebotomus sergenti	58.56223562	38.1468671
Phlebotomus sergenti	58.56223562	38.58706738
Phlebotomus sergenti	55.34996326	39.28900838
Phlebotomus sergenti	54.92166028	37.57579646

species	lon	lat
Phlebotomus sergenti	55.5711	38.134233
Phlebotomus sergenti	56.1470827	38.53947816
Phlebotomus sergenti	56.44451532	39.14624072
Phlebotomus sergenti	57.13455902	39.18193264
Phlebotomus sergenti	57.75321888	38.90829462
Phlebotomus sergenti	57.78891079	38.49188894
Phlebotomus sergenti	56.70625603	38.19445632
Phlebotomus sergenti	56.28183951	27.77638288
Phlebotomus sergenti	54.0974943	28.27606969
Phlebotomus sergenti	57.99505143	27.51940109
Phlebotomus sergenti	60.82185111	26.26304568
Phlebotomus sergenti	61.74662863	28.25108535
Phlebotomus sergenti	67.85708449	29.43129801
Phlebotomus sergenti	70.80761614	32.32472259
Phlebotomus sergenti	71.93072173	32.26761553
Phlebotomus sergenti	67.22018209	30.62658059
Phlebotomus sergenti	66.46728676	30.99849469
Phlebotomus sergenti	66.97033869	31.81631109
Phlebotomus sergenti	62.41367086	31.81631109
Phlebotomus sergenti	63.05612533	31.80441379
Phlebotomus sergenti	63.60340136	32.13753833
Phlebotomus sergenti	62.99663881	32.47066287
Phlebotomus sergenti	62.85387115	33.05363081
Phlebotomus sergenti	62.02105979	33.61280415
Phlebotomus sergenti	62.7467954	33.64849607
Phlebotomus sergenti	62.68730888	34.24336132
Phlebotomus sergenti	63.12750916	34.73115082
Phlebotomus sergenti	64.18636931	31.80441379
Phlebotomus sergenti	64.80502917	31.62595421
Phlebotomus sergenti	65.28092137	31.53077577
Phlebotomus sergenti	65.98286237	31.28093237
Phlebotomus sergenti	65.78060818	31.55457038
Phlebotomus sergenti	66.08993811	31.91148953
Phlebotomus sergenti	66.30408961	32.89896585
Phlebotomus sergenti	66.70859798	33.44624188
Phlebotomus sergenti	65.37609981	34.26715593
Phlebotomus sergenti	66.42306266	34.49320472
Phlebotomus sergenti	67.22018209	34.4456155
Phlebotomus sergenti	67.66038238	34.10059366
Phlebotomus sergenti	68.10058266	34.46941011
Phlebotomus sergenti	67.7079716	32.86327393
Phlebotomus sergenti	68.32663146	32.81568471
Phlebotomus sergenti	69.08805898	32.93465776
Phlebotomus sergenti	69.6177	33.065267
Phlebotomus sergenti	69.17134012	33.42244727
Phlebotomus sergenti	69.21892934	33.93403139

species	lon	lat
Phlebotomus sergenti	68.659756	33.98162061
Phlebotomus sergenti	68.73113983	34.38612898
Phlebotomus sergenti	74.54396478	19.56887829
Phlebotomus sergenti	70.63213089	22.21008001
Phlebotomus sergenti	74.10138503	24.77989789
Phlebotomus sergenti	75.64327576	25.97914624
Phlebotomus sergenti	76.3428373	26.37889569
Phlebotomus sergenti	76.97101501	26.86430573
Phlebotomus sergenti	78.5700128	25.99342301
Phlebotomus sergenti	79.06969961	26.66443101
Phlebotomus sergenti	77.58491595	27.40682284
Phlebotomus sergenti	78.48435221	27.66380463
Phlebotomus sergenti	77.88472803	28.3919197
Phlebotomus sergenti	78.85554812	28.80594591
Phlebotomus sergenti	77.29938062	29.17714183
Phlebotomus sergenti	78.27020072	29.63399834
Phlebotomus sergenti	76.87598528	30.61134212
Phlebotomus sergenti	76.95212803	31.32518043
Phlebotomus sergenti	75.9289598	31.22048414
Phlebotomus sergenti	75.96227226	30.31153004
Phlebotomus sergenti	74.43227883	41.39496026
Phlebotomus sergenti	73.03	40.8513
Phlebotomus sergenti	73.76127083	40.18143515
Phlebotomus sergenti	72.29076392	39.75313216
Phlebotomus sergenti	71.86246094	40.58118459
Phlebotomus sergenti	71.14862264	40.5383543
Phlebotomus sergenti	68.77867948	43.10817218
Phlebotomus sergenti	66.99408372	44.30742053
Phlebotomus sergenti	65.60923742	45.20685679
Phlebotomus sergenti	62.45407213	46.02063245
Phlebotomus sergenti	64.35288201	43.26521661
Phlebotomus sergenti	64.93822942	42.66559244
Phlebotomus sergenti	63.93885579	42.50854801
Phlebotomus sergenti	69.60673191	40.4526937
Phlebotomus sergenti	67.06546755	40.26709574
Phlebotomus sergenti	68.37893003	39.81023923
Phlebotomus sergenti	69.00710774	39.35338272
Phlebotomus sergenti	69.62100868	39.16778476
Phlebotomus sergenti	70.2048	39.598767
Phlebotomus sergenti	70.43478434	39.06784739
Phlebotomus sergenti	70.96302468	39.08212416
Phlebotomus sergenti	71.46271149	39.03929386
Phlebotomus sergenti	71.89101447	38.65382118
Phlebotomus sergenti	72.01950537	37.95425964
Phlebotomus sergenti	70.4918914	38.55388382
Phlebotomus sergenti	68.0048	38.793833

species	lon	lat
Phlebotomus sergenti	67.69364526	39.39621301
Phlebotomus sergenti	67.17968168	39.49615038
Phlebotomus sergenti	69.37830365	38.51105352
Phlebotomus sergenti	69.8208834	38.06847377
Phlebotomus sergenti	68.80723301	38.03992024
Phlebotomus sergenti	68.19333207	37.56878696
Phlebotomus sergenti	69.00710774	37.58306373
Phlebotomus sergenti	66.123201	38.83941914
Phlebotomus sergenti	66.63716457	38.79658884
Phlebotomus sergenti	66.28024542	38.25407173
Phlebotomus sergenti	66.794209	38.21124143
Phlebotomus sergenti	66.558	37.880333
Phlebotomus sergenti	67.15112815	37.95425964
Phlebotomus sergenti	61.21199348	42.40861065
Phlebotomus sergenti	60.26972692	42.0088612
Phlebotomus sergenti	60.8693511	41.52345115
Phlebotomus sergenti	61.59746616	40.73822902
Phlebotomus sergenti	63.33923162	40.75250579
Phlebotomus sergenti	64.83829205	40.26709574
Phlebotomus sergenti	65.10955061	39.66747157
Phlebotomus sergenti	62.49475	39.945033
Phlebotomus sergenti	53.63341018	43.1297063
Phlebotomus sergenti	57.02176265	41.08099038
Phlebotomus sergenti	57.53572623	41.93759634
Phlebotomus sergenti	59.10617049	40.03878646
Phlebotomus sergenti	59.5099	42.313567
Phlebotomus sergenti	61.2476854	38.75387751
Phlebotomus sergenti	60.51957033	37.25481708
Phlebotomus sergenti	61.2476854	37.01211206
Phlebotomus sergenti	61.2476854	37.82588772
Phlebotomus sergenti	61.63315808	37.4975221
Phlebotomus sergenti	61.50466718	36.08412227
Phlebotomus sergenti	62.86095996	35.58443546
Phlebotomus sergenti	63.80322652	35.37028396
Phlebotomus sergenti	64.16014567	35.08474864
Phlebotomus sergenti	63.48913766	34.92770422
Phlebotomus sergenti	63.56052149	36.28399699
Phlebotomus sergenti	62.96089732	37.02638882
Phlebotomus sergenti	62.44693374	37.46896857
Phlebotomus sergenti	63.21787911	37.59745947
Phlebotomus sergenti	63.41775383	38.15425334
Phlebotomus sergenti	63.73184269	38.69677045
Phlebotomus sergenti	63.29175	39.553533
Phlebotomus sergenti	63.83178005	40.11017029
Phlebotomus sergenti	64.44568099	39.39633199
Phlebotomus sergenti	64.53134158	38.72532398

species	lon	lat
Phlebotomus sergenti	64.55989512	38.18280687
Phlebotomus sergenti	64.63127895	37.52607564
Phlebotomus sergenti	65.15951929	37.96865538
Phlebotomus sergenti	65.81625053	37.81161096
Phlebotomus sergenti	65.84775	37.483133
Phlebotomus sergenti	65.53071521	36.54097878
Phlebotomus sergenti	65.01675163	35.88424754
Phlebotomus sergenti	66.34875	37.411467
Phlebotomus sergenti	66.9032	37.426767
Phlebotomus sergenti	67.53965	37.254
Phlebotomus sergenti	66.45013894	36.55525555
Phlebotomus sergenti	67.33529843	36.48387172
Phlebotomus sergenti	67.92064584	36.36965759
Phlebotomus sergenti	68.17762763	36.01273844
Phlebotomus sergenti	68.5928	37.187267
Phlebotomus sergenti	69.20555478	36.8693444
Phlebotomus sergenti	68.64876091	36.65519291
Phlebotomus sergenti	68.83435887	36.29827376
Phlebotomus sergenti	69.19127802	36.26972022
Phlebotomus sergenti	69.60530423	36.52670201
Phlebotomus sergenti	69.84800926	36.08412227
Phlebotomus sergenti	69.59102747	37.25481708
Phlebotomus sergenti	70.1085	37.5722
Phlebotomus sergenti	70.06216075	36.9121747
Phlebotomus sergenti	70.27631224	36.51242525
Phlebotomus sergenti	70.67606169	36.04129197
Phlebotomus sergenti	70.38148441	37.24910637
Phlebotomus sergenti	70.69557327	37.04923165
Phlebotomus sergenti	71.19526008	36.89218722
Phlebotomus sergenti	68.58261189	35.56444798
Phlebotomus sergenti	69.09657547	35.94992067
Phlebotomus sergenti	69.06802194	35.67866211
Phlebotomus sergenti	69.38211079	35.46451062
Phlebotomus sergenti	71.82343778	34.95054704
Phlebotomus sergenti	73.3612	34.4382
Phlebotomus sergenti	74.50984926	34.36282017
Phlebotomus sergenti	75.06902259	34.74353394
Phlebotomus sergenti	75.24748217	35.20752883
Phlebotomus sergenti	75.48542827	34.434204
Phlebotomus sergenti	75.23558486	33.86313336
Phlebotomus sergenti	74.56933578	33.69657109
Phlebotomus sergenti	72.98699421	33.35154925
Phlebotomus sergenti	72.58248584	33.87503067
Phlebotomus sergenti	71.90433946	34.10107946
Phlebotomus sergenti	71.60690683	34.05349024
Phlebotomus sergenti	71.22619307	34.02969563

species	lon	lat
Phlebotomus sergenti	70.78599278	33.63708457
Phlebotomus sergenti	71.39275534	34.434204
Phlebotomus sergenti	71.0715281	35.39788571
Phlebotomus sergenti	71.04773349	34.81491777
Phlebotomus sergenti	70.48856016	33.10170584
Phlebotomus sergenti	71.0596308	33.07791123
Phlebotomus sergenti	68.26376412	35.04096656
Phlebotomus sergenti	68.85862937	35.14804231
Phlebotomus sergenti	68.68016979	34.73163663
Phlebotomus sergenti	69.33452157	35.00527465
Phlebotomus sergenti	69.78661916	34.8863016
Phlebotomus sergenti	70.07215448	34.6602528
Phlebotomus sergenti	70.09594909	34.95768543
Phlebotomus sergenti	70.36958711	34.52938245
Phlebotomus sergenti	70.38148441	34.20815521
Phlebotomus sergenti	70.79789009	34.35092287
Phlebotomus sergenti	70.5956359	35.08855578
Phlebotomus sergenti	68.56119674	35.06476117
Phlebotomus sergenti	69.77472186	34.62456089
Phlebotomus sergenti	74.08154628	31.87107835
Phlebotomus sergenti	38.49216613	8.649045928
Phlebotomus sergenti	39.11082599	9.172527349
Phlebotomus sergenti	-7.711017951	12.74528805
Phlebotomus sergenti	44.7568846	41.7095991
Phlebotomus sergenti	44.7826472	41.6935204
Phlebotomus sergenti	35.6255	37.362689
Phlebotomus sergenti	26.3323237	38.3233928
Phlebotomus sergenti	5.36978	43.296482
Phlebotomus sergenti	-1.1306544	37.9922399
Phlebotomus sergenti	-1.1328244	37.9419513
Phlebotomus sergenti	-2.467504	36.834775
Phlebotomus sergenti	-3.5985571	37.1773363
Phlebotomus sergenti	-5.6635397	40.9701039
Phlebotomus sergenti	-3.7037902	40.4167754
Phlebotomus sergenti	0.810952	41.2133495
Phlebotomus sergenti	0.8454749	41.2344554
Phlebotomus sergenti	2.1734035	41.3850639
Phlebotomus sergenti	1.5208624	41.5911589
Phlebotomus sergenti	-16.6291304	28.2915637
Phlebotomus sergenti	-7.747452	41.299773
Phlebotomus sergenti	-7.9093074	38.5707119
Phlebotomus sergenti	-7.449395	37.179432
Phlebotomus sergenti	-9.1393366	38.7222524
Phlebotomus sergenti	-7.930834	37.0179538
Phlebotomus sergenti	-8.020216	37.1379187
Phlebotomus sergenti	4.2040933	36.6372932

species	lon	lat
Phlebotomus sergenti	10.3385624	33.0641739
Phlebotomus simici	26.3323237	38.3233928
Phlebotomus simici	21.69665	37.3702
Phlebotomus simici	21.20362921	40.40866089
Phlebotomus simici	21.1737	39.073467
Phlebotomus simici	18.08653529	43.28780871
Phlebotomus simici	19.01452508	43.76370091
Phlebotomus simici	19.68077416	43.59713864
Phlebotomus simici	20.41840708	43.52575481
Phlebotomus simici	21.08465616	43.47816559
Phlebotomus simici	21.8936729	43.78749552
Phlebotomus simici	21.72711063	43.21642488
Phlebotomus simici	22.46075	43.653967
Phlebotomus simici	20.48979091	42.88330034
Phlebotomus simici	21.03706694	42.88330034
Phlebotomus simici	20.18375	42.5439
Phlebotomus simici	20.91809389	41.88392672
Phlebotomus simici	21.178105	42.314711
Phlebotomus simici	21.70331602	42.09807821
Phlebotomus simici	21.25121843	41.50321296
Phlebotomus simici	22.03644056	41.57459679
Phlebotomus simici	22.34577049	41.95531055
Phlebotomus simici	22.360773	42.369484
Phlebotomus simici	21.67952141	42.69294346
Phlebotomus simici	22.42695	42.8726
Phlebotomus simici	21.97705	44.6474
Phlebotomus simici	22.61	44.3831
Phlebotomus simici	30.64385	37.0095
Phlebotomus simici	34.05271864	36.95844243
Phlebotomus simici	36.0990551	35.41179278
Phlebotomus simici	36.21802815	33.29407249
Phlebotomus simici	22.96	40.602833
Phlebotomus simici	23.1317	37.821233
Phlebotomus simici	23.7972417	38.14817294
Phlebotomus simici	23.45459932	38.27666383
Phlebotomus simici	23.7972417	38.66689544
Phlebotomus simici	23.65447404	38.30521736
Phlebotomus simici	22.99774281	37.78173594
Phlebotomus similis	19.7431234	41.3715563
Phlebotomus similis	26.46472441	44.60821414
Phlebotomus similis	27.00010314	43.06156449
Phlebotomus similis	22.19575	44.5569
Phlebotomus similis	21.40836978	43.41848364
Phlebotomus similis	22.35445	43.813467
Phlebotomus similis	22.21946855	43.05323638
Phlebotomus similis	21.34858582	42.69631723

species	lon	lat
Phlebotomus similis	21.96248676	42.21090718
Phlebotomus similis	21.46279995	41.62555977
Phlebotomus similis	22.43362004	40.89744471
Phlebotomus similis	32.31433186	47.91566494
Phlebotomus similis	33.28991087	48.0584326
Phlebotomus similis	34.02754379	47.5825404
Phlebotomus similis	14.182839	32.04466003
Phlebotomus similis	33.083	45.848967
Phlebotomus similis	33.6924	45.934967
Phlebotomus similis	34.76041778	45.75987327
Phlebotomus similis	33.9228475	45.26494538
Phlebotomus smirnovi	83.65284411	46.30438387
Phlebotomus smirnovi	80.70707139	43.86424661
Phlebotomus smirnovi	77.40437951	43.86424661
Phlebotomus smirnovi	76.25272039	44.25447821
Phlebotomus smirnovi	75.3783	42.875867
Phlebotomus smirnovi	74.30156236	40.8566079
Phlebotomus smirnovi	61.66376907	37.33500561
Phlebotomus smirnovi	61.58762632	36.44032827
Phlebotomus smirnovi	63.01530292	37.33500561
Phlebotomus smirnovi	63.90998026	36.78297065
Phlebotomus smirnovi	65.40025	38.437567
Phlebotomus smirnovi	68.7831164	37.75379074
Phlebotomus smirnovi	69.34175	40.779267
Phlebotomus smirnovi	68.66890227	42.26524881
Phlebotomus smirnovi	67.67904649	43.63581835
Phlebotomus smirnovi	66.5559409	44.34013881
Phlebotomus smirnovi	65.88969181	44.64470982
Phlebotomus smirnovi	65.31862117	45.06349495
Phlebotomus smirnovi	63.77673044	45.61552991
Phlebotomus smirnovi	62.8820531	45.92010092
Phlebotomus smirnovi	62.17773264	41.57996404
Phlebotomus smirnovi	61.07285	41.296933
Phlebotomus smirnovi	60.40741366	41.54189267
Phlebotomus smirnovi	59.97865	42.042133
Phlebotomus smirnovi	60.90234155	43.02667633
Phlebotomus smirnovi	60.95944861	42.36042725
Phlebotomus smirnovi	60.23609246	42.79824808
Phlebotomus smirnovi	59.30334375	43.00764064
Phlebotomus smirnovi	57.78048871	43.38835441
Phlebotomus transcaucasicus	49.45660564	36.03100724
Phlebotomus transcaucasicus	44.99559215	35.91679311
Phlebotomus transcaucasicus	43.72495997	36.67346171
Phlebotomus transcaucasicus	42.34011366	36.78767584
Phlebotomus transcaucasicus	44.26747708	40.51391177
Phlebotomus transcaucasicus	44.66722653	40.15699262

species	lon	lat
Phlebotomus transcaucasicus	45.34815	39.140567
Phlebotomus transcaucasicus	46.21107919	38.9504915
Phlebotomus transcaucasicus	46.89440203	39.41460079
Phlebotomus transcaucasicus	46.46609905	39.92856436
Phlebotomus transcaucasicus	48.51243551	38.92443182
Phlebotomus transcaucasicus	47.63679386	39.41935971
Phlebotomus transcaucasicus	47.03835942	39.85837026
Phlebotomus transcaucasicus	48.73372539	39.56093764
Phlebotomus transcaucasicus	48.0656	39.681233
Phlebotomus transcaucasicus	47.60348141	40.03682984
Phlebotomus transcaucasicus	47.09784595	40.36400573
Phlebotomus transcaucasicus	46.62195374	40.7804114
Phlebotomus transcaucasicus	46.10775	41.170967
Phlebotomus transcaucasicus	45.6849	41.3707
Phlebotomus transcaucasicus	44.80761473	41.79168233
Phlebotomus transcaucasicus	46.08657502	43.21935893
Phlebotomus transcaucasicus	47.51425162	40.63169509
Phlebotomus transcaucasicus	48.81998085	40.95292233
Phlebotomus transcaucasicus	49.02818369	40.09036771
Phlebotomus transcaucasicus	48.4630617	40.06062445
Phleobotomus tobbi	56.84964356	30.76150663
Phleobotomus tobbi	55.7550915	30.38079287
Phleobotomus tobbi	52.35246226	29.57177613
Phleobotomus tobbi	51.23411559	29.69074918
Phleobotomus tobbi	54.44638794	32.61748622
Phleobotomus tobbi	51.68621318	32.40333473
Phleobotomus tobbi	49.47331444	31.30878266
Phleobotomus tobbi	48.59291387	31.78467486
Phleobotomus tobbi	49.99679586	33.49788679
Phleobotomus tobbi	50.782018	34.61623346
Phleobotomus tobbi	51.47206169	35.25868793
Phleobotomus tobbi	52.37625687	34.92556339
Phleobotomus tobbi	52.70938141	34.09275204
Phleobotomus tobbi	47.33179954	34.04516282
Phleobotomus tobbi	48.11702167	34.56864424
Phleobotomus tobbi	48.78327075	35.6631963
Phleobotomus tobbi	48.9022438	34.90176878
Phleobotomus tobbi	45.5611	35.942133
Phleobotomus tobbi	45.01785	36.709833
Phleobotomus tobbi	42.35445	37.172967
Phleobotomus tobbi	44.75246381	40.3745291
Phleobotomus tobbi	44.97055	39.847
Phleobotomus tobbi	45.62334653	39.1657629
Phleobotomus tobbi	46.5372	38.974333
Phleobotomus tobbi	47.32228169	38.92305788
Phleobotomus tobbi	47.39366552	37.62387217

species	lon	lat
Phleobotomus tobbi	49.27819864	37.12418536
Phleobotomus tobbi	50.24901873	36.69588238
Phleobotomus tobbi	45.0094456	41.76413432
Phleobotomus tobbi	45.7073	41.361367
Phleobotomus tobbi	46.39905082	40.94559974
Phleobotomus tobbi	46.79880027	40.58392166
Phleobotomus tobbi	47.29372816	40.2032079
Phleobotomus tobbi	47.57926348	41.07884955
Phleobotomus tobbi	49.23315	41.0315
Phleobotomus tobbi	49.5941	40.706633
Phleobotomus tobbi	49.05925	41.354233
Phleobotomus tobbi	49.32578786	40.46970754
Phleobotomus tobbi	49.04025254	40.59819843
Phleobotomus tobbi	48.71188692	40.84090345
Phleobotomus tobbi	48.19792334	40.45543077
Phleobotomus tobbi	47.81245066	40.09851162
Phleobotomus tobbi	47.5893	39.637033
Phleobotomus tobbi	48.52628896	40.19844898
Phleobotomus tobbi	48.0656	39.681233
Phleobotomus tobbi	49.02597577	39.82725306
Phleobotomus tobbi	48.52628896	39.49888745
Phleobotomus tobbi	48.56911926	38.85643297
Phleobotomus tobbi	35.35557572	32.27345581
Phleobotomus tobbi	36.21218168	34.95748783
Phleobotomus tobbi	36.29715	36.3222
Phleobotomus tobbi	32.72865077	34.97652352
Phleobotomus tobbi	33.4710426	35.12880902
Phleobotomus tobbi	33.12840022	36.76587819
Phleobotomus tobbi	32.37649054	36.71828897
Phleobotomus tobbi	30.91074256	37.06093136
Phleobotomus tobbi	31.49133104	36.94671723
Phleobotomus tobbi	27.409445	39.7982633
Phleobotomus tobbi	25.29095	41.031667
Phleobotomus tobbi	23.67025	40.3528
Phleobotomus tobbi	23.07882597	40.70602767
Phleobotomus tobbi	23.74570958	38.12923003
Phleobotomus tobbi	21.27126842	38.82006688
Phleobotomus tobbi	21.29506303	39.87892703
Phleobotomus tobbi	20.81917083	41.68731739
Phleobotomus tobbi	21.37834417	42.03233924
Phleobotomus tobbi	21.9375175	41.46126859
Phleobotomus tobbi	22.00890133	42.04423654
Phleobotomus tobbi	22.06838786	42.59151257
Phleobotomus tobbi	19.53025	41.902933
Phleobotomus tobbi	18.87991011	42.43684761
Phleobotomus tobbi	18.26125025	42.9008425

species	lon	lat
Phleobotomus tobbi	17.49982273	43.41242662
Phleobotomus tobbi	16.75965662	43.83659007
Phleobotomus tobbi	35.6255	37.362689
Phleobotomus tobbi	26.3323237	38.3233928
Phleobotomus tobbi	19.089904	42.0912106
Phleobotomus tobbi	19.7431234	41.3715563
Phleobotomus tobbi	19.6877385	41.8217526

References

1. Artemiev VM, Neronov VM, editors (1984) Distribution and ecology of sandflies of the Old World (genus *Phlebotomus*). Moscow: The USSR Committee for the Unesco Programme on Man and the Biosphere (MAB) Institute of Evolutionary Morphology and Animal Ecology; USSR Academy of Science.
2. Akhoundi M, Parvizi P, Baghaei A, Depaquit J (2012) The subgenus *Adlerius Nitzulescu* (Diptera, Psychodidae, *Phlebotomus*) in Iran. Acta tropica 122 (1): 7–15.
3. Berdjane-Brouk Z, Charrel RN, Hamrioui B, Izri A (2012) First detection of *Leishmania infantum* DNA in *Phlebotomus longicuspis* Nitzulescu, 1930 from visceral leishmaniasis endemic focus in Algeria. Parasitology Research 111 (1): 419–422. doi:10.1007/s00436-012-2858-1.
4. Boussaa S, Boumezzough A, Remy PE, Glasser N, Pesson B (2008) Morphological and isoenzymatic differentiation of *Phlebotomus perniciosus* and *Phlebotomus longicuspis* (Diptera Psychodidae) in Southern Morocco. Acta tropica 106 (3): 184–189. doi:10.1016/j.actatropica.2008.03.011.
5. Giorgobiani E, Lawyer PG, Babuadze G, Dolidze N, Jochim RC, et al. (2012) Incrimination of *Phlebotomus kandelakii and Phlebotomus balcanicus* as vectors of

Leishmania infantum in Tbilisi, Georgia. PLoS Neglected Tropical Diseases 6 (4): e1609. doi:10.1371/journal.pntd.0001609.

6. Benabdennbi I, Pesson B, Cadi-Soussi M, Marquez FM (1999) Morphological and isoenzymatic differentiation of sympatric populations of *Phlebotomus perniciosus* and *Phlebotomus longicuspis* (Diptera Psychodidae) in Northern Morocco. Journal of Medical Entomology 36 (1): 116–120. doi:10.1093/jmedent/36.1.116.
7. Rassi Y, Javadian E, Nadim A, Rafizadeh S, Zahraii A, et al. (2009) *Phlebotomus perfiliewi transcaucasicus*, a Vector of *Leishmania infantum* in Northwestern Iran. Journal of Medical Entomology 46 (5): 1094–1098. doi:10.1603/033.046.0516.
8. Steinhauser I (2005) Untersuchung zur Verbreitung von Sandmücken (Phlebotomen) in Deutschland mit Hilfe geographischer Informationssysteme (GIS). Diploma thesis. Bonn.
9. Svobodová M, Alten B, Zídková L, Dvořák V, Hlavačková J, et al. (2009) Cutaneous leishmaniasis caused by *Leishmania infantum* transmitted by *Phlebotomus tobbi*. International Journal for Parasitology 39 (2): 251–256.
10. Tabbabi A, Bousslimi N, Rhim A, Aoun K, Bouratbine A (2011) First report on natural infection of *Phlebotomus sergenti* with *Leishmania* promastigotes in the cutaneous leishmaniasis focus in southeastern Tunisia. The American Journal of Tropical Medicine and Hygiene 85 (4): 646–647. doi:10.4269/ajtmh.2011.10-0681.
11. Tarallo VD, Dantas-Torres F, Lia RP, Otranto D (2010) Phlebotomine sand fly population dynamics in a leishmaniasis endemic periurban area in southern Italy. Acta Tropica 116 (3): 227–234.
12. Weise M (2004) Reisetiermedizinisch und epidemiologisch wichtige Arten der kaninen Parasitenfauna in europäischen Anrainerstaaten des Mittelmeeres und in Portugal für Hunde in Deutschland. PhD thesis. Munich.
